# Micrometer-scale indirect photopatterning of RGB OLED emissive layers in single phase network structure

**DOI:** 10.1038/s41377-025-01907-w

**Published:** 2025-07-22

**Authors:** Seunghan Lee, Hyobin Ham, Shahid Ameen, Byung Hak Jhun, SeungHwan Roh, Hyeono Yee, Chang Hyeok Lim, Yuchan Heo, Hyukmin Kweon, Dongheon Han, Do Hwan Kim, Youngmin You, BongSoo Kim, Moon Sung Kang

**Affiliations:** 1 Department of Chemical and Biomolecular Engineering, Sogang University, Seoul, 04107 Republic of Korea; 2 Department of Chemistry, Ulsan National Institute of Science and Technology (UNIST), Ulsan, 44919 Republic of Korea; 3 Department of Chemical and Biomolecular Engineering, Yonsei University, Seoul, 03722 Republic of Korea; 4 Department of Chemical Engineering, Hanyang University, Seoul, 04763 Republic of Korea; 5 Institute of Nano Science and Technology & Clean-Energy Research Institute, Hanyang University, Seoul, 04763 Republic of Korea; 6 Graduate School of Semiconductor Materials and Device Engineering & Graduate School of Carbon Neutrality, Ulsan National Institute of Science and Technology (UNIST), Ulsan, 44919 Republic of Korea; 7 Institute of Emergent Materials, Sogang University, Seoul, 04107 Republic of Korea

**Keywords:** Organic LEDs, Photonic devices

## Abstract

Organic light-emitting diodes (OLEDs) used in virtual and augmented reality displays require micrometer-scale red-green-blue (RGB) pixel patterns in the emissive layer (EML). However, conventional patterning methods based on evaporation and shadow masks can only produce patterns larger than tens of micrometers owing to the geometric constraint of the mask. Herein, an indirect method for photopatterning solution-processed OLED EMLs is proposed, which can be used to form micrometer-scale RGB pixel patterns without involving direct exposure to UV radiation or harsh etching processes on EMLs. EMLs can be patterned by i) forming a sacrificial photoresist (PR) pattern, ii) spin-coating an EML film, iii) converting the EML film into a single-phase network (SPN) structure by crosslinking vinylbenzyl-group-appended hosts and dopants at a low temperature, and iv) stripping the pre-formed PR pattern. Furthermore, repeating the process thrice results in the formation of RGB EML patterns. During the repeated process, the sacrificial PR pattern serves as a protective layer for the underlying EML pattern, effectively preventing the EML pattern from being exposed to solutions in subsequent processes. Using a conventional photolithography setup, we produced sets of RGB EML patterns with densities exceeding 3000 patterns/in., which indicated the potential of the method for industrial use.

## Abstract (web summary)

This work introduces a photolithographic process to form micrometer-scale organic RGB pixel patterns based on molecular crosslinking strategy. The method can be utilized for achieving high-resolution full-color OLEDs.

## Graphical abstract

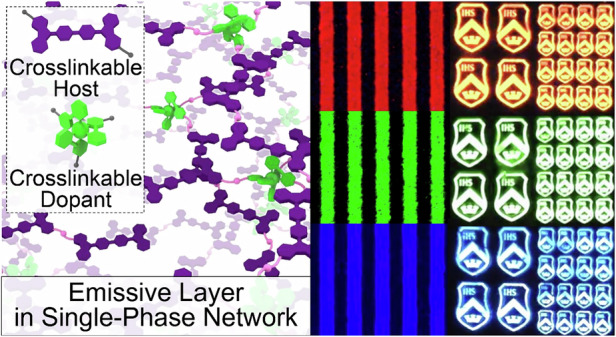

## Introduction

Organic light-emitting diodes (OLEDs) have become one of the most successful display technologies and continue to evolve for next-generation applications^1–7^, including ultrahigh-resolution near-eye displays for virtual/augmented reality (VR/AR) systems^8^. Specifically, micro-OLEDs have emerged as a key technology in the industry, primarily relying on color filters^9^ or advanced color converting optical structures^10^ integrated at an ultrahigh resolution on top of a common white OLED^11^. Despite the advances, however, this approach inherently suffers from optical crosstalk between subpixels, a challenge that becomes increasingly critical as display resolution increases^12–14^. To eliminate optical crosstalk at high resolutions, a self-emissive OLED structure with directly patterned functional layers is required. Among the various functional layers of OLEDs, the patterning of the emissive layer (EML) is particularly crucial for achieving high-resolution full-color display with micrometer-scale subpixels^15–20^. The standard method used for forming large EML patterns in the current display industry is based on thermal evaporation employing a shadow mask. However, the geometry of the mask limits the pixel dimension, which typically exceeds tens of micrometers. Furthermore, the thermal evaporation method is associated with poor pattern fidelity for small features, owing to the shadow region between the substrate and the mask^21,22^. Another drawback of the shadow-mask-based method is the sagging or warping of masks in the manufacture of very large-area display panels. Consequently, there is a need for an alternative to the evaporation-based patterning technique for OLEDs for futuristic VR/AR technology^23–25^.

Photolithography is a workhorse patterning method that is widely used in the semiconductor and display industries. Fabricating patterns of various materials on a micrometer-scale can be readily achieved with exceptional reliability, meeting industrial standards. Therefore, for the formation of patterns on each red-green-blue (RGB) EML in OLEDs, photolithography is a promising method^26–30^. However, the following constraints prevent the direct adaptation of the conventional photolithographic method for the formation of EML patterns. First, organic luminophores are much softer than conventional inorganic semiconductors such as silicon, and hence, etching processes involving reactive and corrosive chemicals (e.g., Ar/O_2_, CF_*x*_, SF_6_) result in patterns with low fidelity (especially with low line edge roughness)^26^. Second, organic luminophores can swell or dissolve in organic solvents used in conventional photolithography^30^. Therefore, a photoresist (PR) layer, which is spin-coated using an organic solvent, should be introduced and removed delicately without affecting the underlying organic luminophore layer. Direct photopatterning of materials based on a light-induced crosslinking reaction of luminophores has recently emerged as a promising method for circumventing the above constraints, as it does not involve the use of a PR layer^31–35^. However, the luminescence characteristics of organic luminophores can be degraded under direct exposure to UV radiation^36–39^. Thus, direct UV irradiation of the material should be avoided or minimised during EML patterning.

Here, we propose the indirect photopatterning of micrometer-scale RGB EMLs in a single-phase network (SPN) structure, that is, a network structure of chemically crosslinked-host and dopant molecules. The term SPN emphasizes that both host and dopant molecules collectively form a network in a single phase, distinguishing it from an interpenetrating network composed of two independent networks, each consisting of either host or dopant molecules, entangled with each other. The SPN structure exhibiting chemical resistance to various solvents can be formed using newly synthesised organic luminophores (both host and dopant) appended with vinylbenzyl groups. The vinylbenzyl groups can be thermally crosslinked at a low temperature (<120 °C; this low processing temperature is what makes indirect photopatterning possible, as explained later). Using these host and dopant molecules, we performed indirect photopatterning of RGB EML by repeating the following steps: (i) forming a sacrificial PR pattern, (ii) spin-coating an EML film comprising host and dopant molecules, (iii) converting the EML into the SPN structure through crosslinking, and (iv) stripping the PR pattern. The dimensions of the resulting EML patterns were determined by the shape of the PR pattern utilised. Notably, the approach enables the formation of EML patterns without direct exposure of the material to UV radiation or any harsh etching process. Additionally, the underlying EML patterns are protected from degradation and contamination during the multicolor EML patterning process. Repeating the proposed indirect photopatterning method, we formed a set of multicolor EML patterns with a density exceeding 3000 RGB patterns/in. We emphasize that micrometer-scale patterns on RGB EMLs can be formed with commercially available PRs and a conventional photolithography setup. The proposed method can be readily adopted by the display industry for the manufacture of micrometer-scale OLED displays.

## Results

### Description of indirect photopatterning of OLED EML

**Fig. 1:**
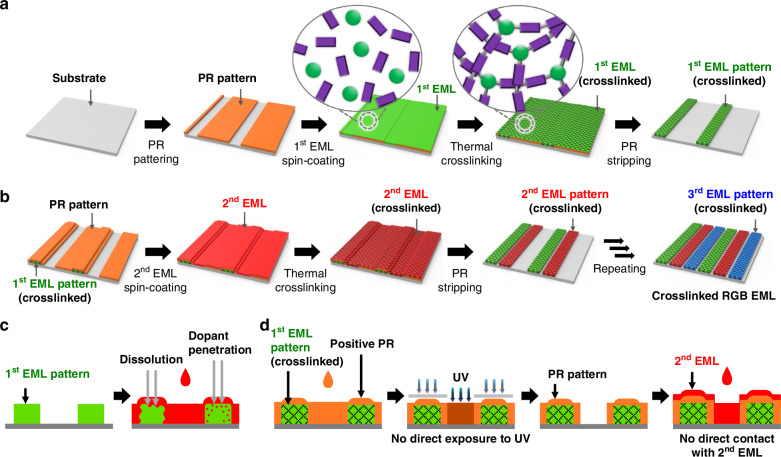
Schematic of indirect photopatterning of RGB EML in the SPN structure. **a** Process flow for the indirect photopatterning of the first EML pattern in the SPN structure. In the circled schematics, green circles and purple rectangles represent hosts and dopants, respectively. **b** Process flow for the indirect photopatterning of the second and third EML patterns in the SPN structure. **c** Side view of the conventional/direct photopatterning steps for the second EML pattern; the steps show the possibility of dissolution and contamination of the underlying EML pattern. **d** Side view of the indirect photopatterning steps for the second EML pattern, emphasizing the absence of both direct UV irradiation of the EML and direct contact of the EML with the subsequently deposited emissive color material

Our indirect photopatterning of an EML in the SPN structure involved the following steps. First, a sacrificial PR pattern was formed using a positive-tone PR, which defined the regions of EMLs to be patterned (Fig. 1a). Subsequently, an EML was directly formed over the PR-patterned substrate by spin-coating a luminophore solution (a mixture of the host and the dopant). The EML was then thermally annealed to induce a crosslinking reaction between the host and dopant molecules, resulting in the formation of the SPN structure. The SPN structure exhibited high chemical resistance in the subsequent solution processes. The formation of the SPN structure is a necessity in the proposed patterning method (the need for an EML with high chemical resistance is discussed later in this section). Finally, the sacrificial PR pattern was removed using a solvent (i.e., PR stripper), resulting in micrometer-scale EML patterns. Notably, this method facilitates the photopatterning of an EML without requiring direct exposure of the light-sensitive EML to UV radiation. This is one of the two important features that are not found in the currently used *direct* photopatterning method, and therefore, we refer to the proposed method as *indirect* photopatterning. The second feature is explained below.

A more significant advantage of using the sacrificial PR pattern is evident when the patterning process is repeated to form RGB patterns in sequence (Fig. 1b). Let us consider the formation of the second color pattern next to the already-formed first-color pattern. If the conventional PR-based photolithographic patterning protocol or direct photopatterning protocol is used, the second color EML should be directly deposited over the first color EML pattern (Fig. 1c). Since the solubilities of the materials used as the first and second color materials are typically similar, the solvent used to cast the second color solution would be one in which the material of the already-formed first color pattern would easily dissolve. Hence, the first color pattern can dissolve in the solvent used for the second color solution or become swollen, or the material of the second color EML can penetrate the underlying first-color pattern and cause color contamination of the luminescence^25,40–43^. In the proposed indirect photopatterning method, the sacrificial PR pattern (used to pre-define the region of the first EML to be patterned) also serves as a protective layer, preventing the direct contact of the second color material with the first color pattern (Fig. 1d). Therefore, if the same sacrificial-PR-assisted patterning method is repeated for the second color material, the second color EML can be patterned next to the first color EML pattern without any dissolution, swelling, or color contamination. Similarly, the third color materials can be used for patterning adjacent to the first and second color EML patterns by repeating the aforementioned patterning steps. This is the other important feature of the proposed patterning method, and it was also a reason for naming the method as *indirect* patterning. Overall, the proposed indirect photopatterning method facilitates the formation of patterns of RGB EMLs without involving (i) direct exposure of the light-sensitive EML materials to UV radiation and (ii) direct exposure of one color pattern to other color materials during subsequent patterning steps.

For the above patterning steps to be successful, we point out that the integrity of the EML must remain unaffected when exposed to a variety of solvents during the subsequent processing steps. The EML patterns should remain unaffected when the sacrificial PR pattern is stripped using a chemical reagent. Furthermore, the EML patterns should not dissolve or swell when the PR layer (used to define the next color pattern) is directly deposited on them. Additionally, they should remain intact during the subsequent coating of the next color EMLs used for producing RGB pixels^44^. All these requirements are fulfilled, as the formation of the SPN structure imparts chemical resistance to the EML.

### Formation of EML in the SPN structure

**Fig. 2:**
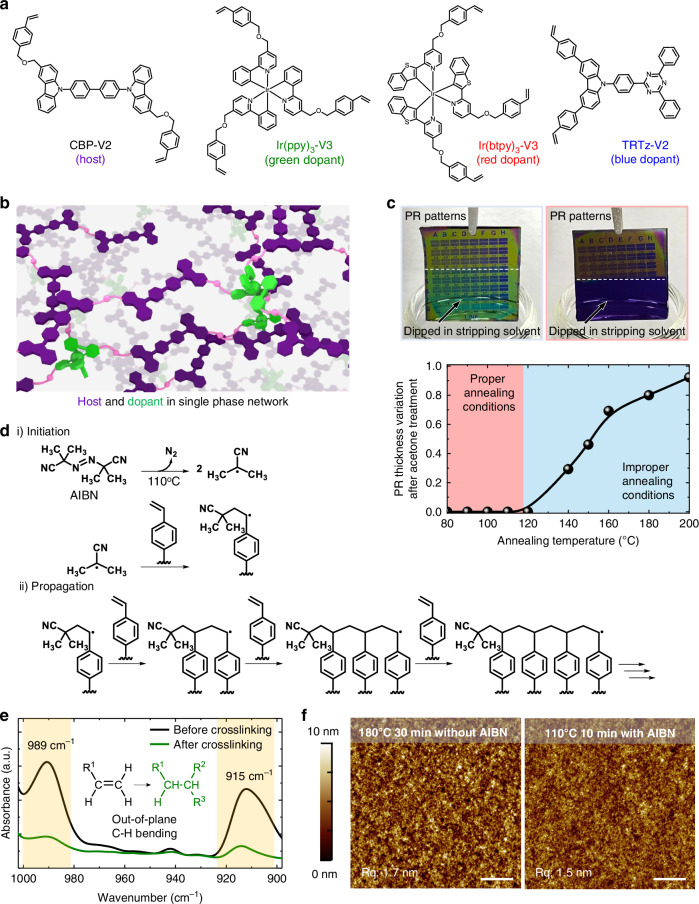
Formation of the SPN structure through low temperature annealing at 110 °C. **a** Chemical structures of crosslinkable organic luminophores. **b** Schematic of the SPN structure with the host and dopant molecules being crosslinked. **c** Photographs of PR patterns (KL5301) after immersion in the stripping solvent; patterns annealed at 180 °C for 30 min (left, blue box) and at 110 °C for 10 min (right, red box) are shown. The panel also shows a plot of the relative thickness of thermally annealed PR films after immersion in acetone (bottom), a stripping solvent, versus the annealing temperature. **d** Mechanisms of initiation of radical polymerisation, which begins with thermal decomposition of AIBN and propagation between vinylbenzyl moieties in the host and the dopant. **e** FT-IR spectra of the green EML film before and after the thermal crosslinking reaction, indicating the near-complete disappearance of the out-of-plane C-H bending mode. **f** AFM topography images of crosslinked green EMLs with different annealing temperatures: 180 °C (left; without AIBN) and 110 °C (right; with AIBN). Scale bars are 2 μm

To form SPN structures with host and dopant molecules, we appended crosslinkable functional groups to both types of molecules. Specifically, we synthesised host and dopant molecules appended with vinylbenzyl groups, which is an approach that has been widely used in various organic semiconductors employed as hole transport layer (HTL) materials^45–47^, host materials^48,49^, and dopants^50–52^. The chemical structures of the host molecule, namely 9,9′-(1,1′-biphenyl)-4,4′-diylbis(3-(((4-ethenylphenyl)methoxy)methyl)-9*H*-carbazole) (CBP-V2), and the dopant molecules—green-emitting *fac*-tris(2-(3-(((4-vinylbenzyl)oxy)methyl)phenyl)pyridine)iridium(III) (Ir(ppy)_3_-V3), red-emitting *mer*-tris(2-(benzo[*b*]thiophen-2-yl)-4-(((4-vinylbenzyl)oxy)methyl)pyridine)iridium(III) (Ir(btpy)_3_-V3), and blue-emitting 9-(4-(4,6-diphenyl-1,3,5-triazin-2-yl)phenyl)-3,6-bis(4-vinylphenyl)-9*H*-carbazole (TRTz-V2)—are shown in Fig. 2a. Detailed synthesis methods for these molecules and their photophysical properties are provided in Supplementary Information. Each of the RGB EMLs prepared in this study consisted of one of these color-emitting dopants and the host.

Using these crosslinkable molecules, an EML in the SPN structure could be simply formed by spin-coating a host-dopant mixture, followed by thermal annealing above 180 °C to promote the radical polymerisation of the appended vinylbenzyl groups (Fig. 2b)^48,50,53–56^. The progress of the reaction was confirmed by the disappearance of the vibration peak in the FT-IR spectrum for the mixture film after the annealing. Importantly, the resulting EML film showed good chemical resistance to various solvents (Fig. S2 and Fig. S3).

Despite the successful crosslinking, annealing at 180 °C altered the chemical/structural properties of the PR layer that we used. For example, the photograph in Fig. 2c (left) shows the PR pattern (KL5301, KemLab) on a Si/SiO_2_ wafer thermally annealed at 180 °C for 30 min. Despite the immersion of the PR films in acetone, the stripping solvent for KL5301, the PR pattern remained perfectly intact, indicating that the physical properties of the KL5301 were altered by the 180 °C annealing and that it could not be stripped after the annealing. We scanned annealing temperatures (for a short duration of 10 min) to determine an optimal condition for crosslinking vinyl groups without affecting the physical properties of the PR films. The variation of the PR film thickness with the annealing temperature is shown in Fig. 2c; the thicknesses were measured before and after immersion in acetone. The results indicate that the PR film can be removed completely using the given stripping solvent only when the annealing temperature is below 130 °C; for instance, the photograph in Fig. 2c (right) shows the KL5301 PR pattern formed on an Si/SiO_2_ wafer annealed at 110 °C; the pattern was completely removed upon immersion in the stripping solvent. This implies that the annealing temperature required to induce thermal crosslinking in the EML should be below 130 °C for KL5301. Accordingly, we introduced a low temperature thermal initiator, namely 2,2′-azobis(2-methylpropionitrile) (AIBN) in the EML (Fig. 2d). AIBN is known to be activated at temperatures above 60 °C^57,58^, and its activation would generate free radicals that initiate the crosslinking reaction of vinylbenzyl groups in the host and dopant (Fig. 2d). For example, Fig. 2e shows the FT-IR spectra of the green EML film composed of Ir(ppy)_3_-V3, CBP-V2, and AIBN (12: 87: 1 wt%) that were obtained before and after annealing at 110 °C for 10 min. Annealing at 110 °C resulted in the near-complete removal of the peak intensity (989 and 915 cm^−1^) associated with out-of-plane C-H bending in the vinyl groups, which confirmed the formation of the SPN structure (Fig. 2e). Similar to the green EML, FT-IR spectra of the red (composed of Ir(btpy)_3_-V3, CBP-V2, and AIBN in 12: 87: 1 wt% ratio) EML and blue (composed of TRTz-V2, CBP-V2, and AIBN in 10: 89: 1 wt% ratio) EML also showed the reduction in peak intensity near 989 and 915 cm^−1^ after annealing at 110 °C for 10 min, indicating the formation of the SPN structure (Fig. S4). Moreover, the low temperature crosslinking yielded films with the desired chemical resistance. Fig. S5 shows the UV-Vis spectra of the crosslinked R/G/B EML films in the SPN structure; the spectra were recorded before and after rinsing with the mother solvent (i.e., chloroform or chlorobenzene) that was used to cast the original film. Despite the immersion of the films in the mother solvent, the shape and intensity of the spectrum remained unchanged, indicating that the crosslinked films were resistant to the mother solvent. Figure 2f shows the atomic force microscopy (AFM) morphology of the EML film in the SPN structure under different conditions; the left image corresponds to a green EML crosslinked at 180 °C for 30 min without AIBN, while the right image pertains to a sample crosslinked at 110 °C for 10 min with AIBN. There is no significant difference in the film morphology, but the EML film crosslinked at 110 °C exhibits a slightly lower surface roughness (root mean square (RMS) roughness: 1.5 nm) than the high temperature crosslinked EML film (RMS roughness: 1.8 nm); a smoother surface is more beneficial for the formation of subsequent layers in OLED fabrication.

### Characteristics of RGB EML patterns

**Fig. 3:**
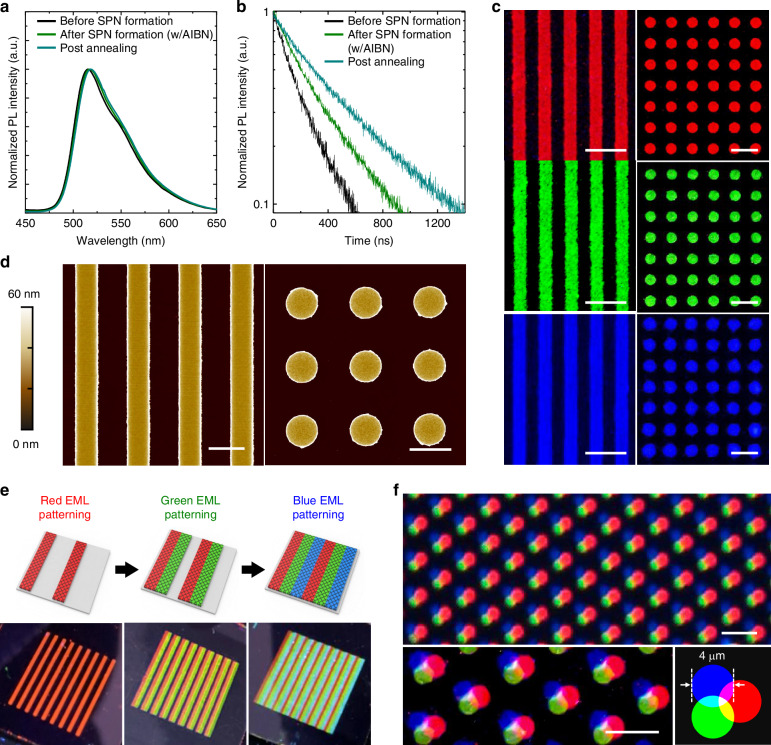
PL properties of an EML film in the SPN structure and high-resolution mono-/multicolor patterns. Variation of the **a** PL spectra and **b** transient PL spectra with annealing conditions. **c** Fluorescence microscopy images of line-shaped (width: 3 µm; spacing: 4 µm) and circular (width: 4 µm; spacing: 4 µm) R/G/B patterns. The scale bar is 10 μm. **d** AFM topography images of line-shaped (width: 3 µm; spacing: 4 µm) and circular (width: 4 µm; spacing: 4 µm) green EML patterns. The scale bar is 5 μm. **e** Schematic images of the RGB multicolor line-patterning process and photographs of the patterns under UV exposure. **f** Fluorescence microscopy images depicting the three primary colors of light through R/G/B patterns (diameter of circles: 4 µm) and a schematic image (bottom right). The scale bar is 10 μm

Next, photoluminescence (PL) characteristics of the EML film in the SPN structure were examined. The black and green curves in Fig. 3a show a comparison of the normalized PL spectra of the green EML film composed of Ir(ppy)_3_-V3, CBP-V2, and AIBN (12:87:1 wt%) that were obtained before and after annealing at 110 °C for 10 min, respectively. After the SPN structure formation, the EML film showed a small red-shift (2 nm) in the PL spectra but without a significant change in the overall shape. The red-shift of the PL spectra was consistently observed for red/blue EMLs after the SPN structure formation (Fig. S6), which can be attributed to the shortening of intermolecular distances between the luminophores because of crosslinking^59^. Despite the reduced intermolecular distance, the PL quantum yield (PLQY) of the film surprisingly increased from 9.2% to 16.9% after the SPN structure formation (Fig. S7). Interestingly, the PLQY of the film increased even more when the film was post-annealed at higher temperatures (e.g., PLQY = 19.8% after annealing at 180 °C for 30 min). To understand the reason for the higher PLQY after crosslinking, we examined the transient PL characteristics (Fig. 3b). The average exciton lifetime (*τ*) of the films was obtained from the time taken for the PL intensity to decay to 1/e of the initial intensity. The parameter *τ* increased from 0.61 µs to 0.95 µs after the SPN structure formation, and it further increased up to 1.4 µs after post-annealing at 180 °C. These results indicate that the luminescence characteristics of the EML pattern (formed through annealing at 110 °C) can be further enhanced by the subsequent post-annealing step at 180 °C after the patterning process. We conjecture that the small amount of unreacted vinylbenzyl groups in the EML served as a quenching site for excitons and that the post-annealing steps facilitated the completion of the crosslinking reactions involving the unreacted vinylbenzyl groups. Furthermore, we examined changes in the PL properties of EMLs under three rinsing cycles, as three rinsing cycles represent the minimum number of rinses an EML film may undergo in our patterning process. Although the non-normalized PL spectra showed a reduction in intensity due to the decrease in film thickness after the first rinsing (Fig. S8), there was no significant change in the overall spectral shape after both the first rinsing and the subsequent three rinsing cycles (Fig. S9). More importantly, the PLQY of the R/G/B EML films showed no significant change during the three rinsing cycles. Therefore, we conclude that the PL properties of the EML films are preserved during our proposed indirect photopatterning method. Transient PL spectra of red/blue EMLs at different conditions were also monitored (Fig. S10). Unlike green EML, the *τ* values attained from red/blue EMLs gradually decreased after applying the annealing step. Further investigation is needed to understand the different influences of crosslinking on luminescence properties.

Using the proposed indirect photopatterning method, we successfully patterned R/G/B EMLs (Fig. 3c). Importantly, our patterning method enabled the formation of micrometer-scale patterns; line patterns with a width of 3 µm and circular patterns with a diameter of 4 µm were successfully formed on R/G/B EMLs (Fig. 3c). Large-area OM images of line patterns without UV radiation are displayed in Fig. S11. The surface morphology of the two patterns was obtained from AFM measurements, and it is shown in Fig. 3d. In addition, due to the optimal crosslinking reaction temperature that did not alter the solubility characteristics of the PR, the EML patterns were formed without residues (Fig. S12). These results show that our patterning method is a promising route for producing high-resolution micrometer-pixelated OLEDs. More importantly, we successfully prepared side-by-side line-patterned RGB EMLs (Fig. 3e) by repeating the indirect patterning method, as described in Fig. 1. It should be emphasized that the chemical resistance of the EML in the SPN structure facilitated the repeated application of the patterning processes for the different color EMLs adjacent to pre-formed patterns; that is, if the pre-formed EML patterns had not been crosslinked, they would have readily dissolved during the patterning steps of the second/third color EMLs. Additionally, ‘the three primary colors of light’ with a diameter of 4 µm were formed by sequentially patterning R/G/B EMLs, and the formation was confirmed from a fluorescence microscopy image (Fig. 3f). The results showed that crosslinked EMLs could not only be formed adjacent to each other laterally but also stacked vertically. More than 3000 sets of circular RGB patterns were successfully formed. The results showed that through the indirect photopatterning method, RGB pixel patterns with a higher resolution can be achieved compared with those that can be obtained through thermal evaporation patterning with a shadow mask.

### EL characteristics of R/G/B OLEDs

**Fig. 4:**
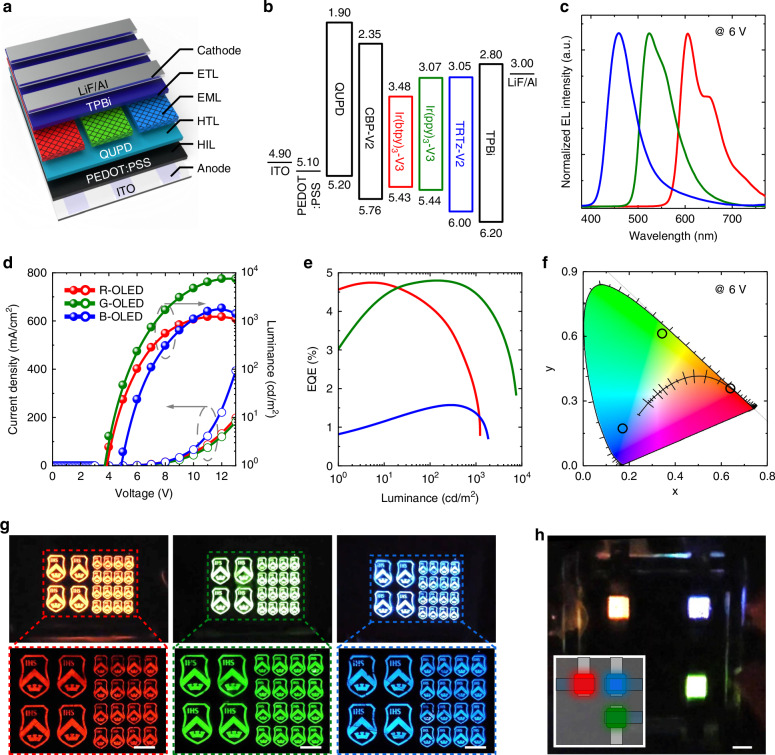
EL characteristics of R/G/B OLEDs with EMLs in the SPN structure. **a** Device structure scheme of OLEDs. **b** Energy level diagram of OLED layers. **c** EL spectra, **d** *J*-*V*-*L* curves, and **e** EQE curves of R/G/B OLEDs. **f** CIE plot of R/G/B OLEDs. **g** Photographs and EL images of operating patterned OLEDs. The scale bar is 2 mm. **h** A photograph of an operating multicolor patterned OLED. The scale bar is 2 mm

Finally, we examined the electroluminescence (EL) characteristics of the EMLs in the SPN structure for ascertaining the suitability of EMLs prepared through the proposed method for optoelectronic devices. For R/G/B EMLs, we used Ir(ppy)_3_-V3 (12 wt%) for green emission, Ir(btpy)_3_-V3 (12 wt%) for red emission, and TRTz-V2 (10 wt%) for blue emission, along with the CBP-V2 host and the thermal initiator (AIBN, 1 wt%). OLEDs were fabricated in a conventional bottom-emission structure, as shown in Fig. 4a^60^. In these devices, poly(3,4-ethylenedioxythiophene) polystyrene sulfonate (PEDOT:PSS) and *N*,*N*′-bis(4-(6-((3-ethyloxetan-3-yl)-methoxy)-hexyloxy)phenyl)-*N*,*N*′-bis(4-methoxyphenyl)biphenyl-4,4′-diamine (QUPD) were used as the hole injection layer (HIL) and hole transport layer (HTL), respectively. Notably, the given HTL material contained oxetane groups that promoted living polymerisation via cations of PEDOT:PSS^61^, which prevented the underlying HTL from being damaged during the deposition and patterning of the EMLs. 2,2′,2′′-(1,3,5-Benzinetriyl)-tris(1-phenyl-1-H-benzimidazole) (TPBi) and LiF/Al were used as the electron transport layer (ETL) and cathode, respectively. Figure 4b shows the energy band diagrams of the OLED layers. The energy levels of the constituent layers of the OLEDs were calculated using cyclic voltammetry (CV) and Tauc plots (Fig. S13 and Fig. S14).

The as-fabricated R/G/B OLEDs showed EL spectra peaks at 460 nm for blue, 524 nm for green, and 604 nm for red (Fig. 4c). Figure 4d, e show the EL performance of the R/G/B OLEDs. The green OLED exhibited a turn-on voltage of 3.7 V, a maximum luminance of 7581 cd/m^2^, and a maximum external quantum efficiency (EQE) of 4.8%. The red OLED had a similar turn-on voltage of 3.9 V and showed a maximum luminance of 1233 cd/m^2^ and a maximum EQE of 4.9%. The blue OLED exhibited a slightly higher turn-on voltage of 4.9 V, a maximum luminance of 1866 cd/m^2^, and a maximum EQE of 1.6%. Detailed data for the R/G/B OLEDs are presented in Supplementary Table 2. Note that these results are superior to previous R/G/B OLEDs utilizing luminophores with the same crosslinkable group (Supplementary Tables 3–5). While the performance of our current devices is lower than that of OLEDs fabricated via evaporation using the same luminophore core, we believe this is primarily because these luminophores were more suitable for evaporation-based processes. However, if luminophore cores specifically designed for solution processing^62–64^ were modified with crosslinkable functional groups^65^, further performance enhancements could be achieved. Positions of the EL spectra in the Commission Internationale de l'éclairage (CIE) coordinates are shown in Fig. 4f. At an operating voltage of 6 V, the R/G/B OLEDs showed values of (0.17, 0.17) for blue, (0.35, 0.61) for green, and (0.64, 0.36) for red.

To verify the applicability of the proposed patterning method for OLEDs, we fabricated OLEDs with a patterned EML (featuring the emblem of Sogang University) and obtained photographs during the operation of the OLEDs (Fig. 4g). The devices exhibited appropriate luminescence in the patterned areas. The detailed structure of the OLEDs patterned in the shape of the emblem is shown in Fig. S15. More importantly, we fabricated OLEDs with patterned R/G/B EMLs by repeating the patterning process thrice on a single substrate. This was to examine the feasibility of the proposed method for full-color display applications. Through repeated cycles, we successfully fabricated OLEDs with individual R/G/B color emission patterns. The photograph in Fig. 4h was taken when the R/G/B OLEDs were operated simultaneously. These results indicate that our patterning method can be applied to full-color display applications without adversely affecting the optoelectronic properties of EMLs. The detailed fabrication process is shown in Fig. S16.

## Discussion

An indirect photopatterning method was developed for the preparation of high-resolution OLED EMLs. The method involves two key strategies. First, from a procedural standpoint, the method exploits pre-formed sacrificial PR patterns that define the dimensions of the EML. The use of PR patterns allows the indirect formation of the EML pattern, and there is no need for direct exposure to UV radiation or harsh etching processes. Additionally, the PR layer prevents color contamination during subsequent steps of patterning other color layers, which is important when generating multicolor pixels. Second, from a materials perspective, the success of indirect photopatterning is dependent on employing an EML in the SPN structure formed at a low temperature. The SPN structure provides chemical resistance against various solvents used in later patterning steps, while its mild crosslinking temperature permits the complete stripping of sacrificial PR layers in the final patterning step. We synthesised R/G/B-emitting luminophores and host molecules appended with multiple vinylbenzyl groups, which could undergo thermal crosslinking at 110 °C in the presence of an AIBN initiator to form the SPN structure. Based on all these strategies, we successfully fabricated EML patterns on a 3 µm scale. Importantly, we formed RGB full-color EML patterns by repeating the patterning method with R/G/B color luminophores. We emphasize that the entire process was conducted using a conventional photolithography setup. The proposed method holds significant promise for realising micrometer-scale high-resolution RGB OLED displays.

## Materials and methods

### Materials for synthesis

Deuterated chloroform (CDCl_3_) and deuterated dimethyl sulfoxide (DMSO-*d*_6_) for measurement of ^1^H-NMR and ^13^C-NMR spectra were purchased from Cambridge Isotope Laboratories (USA). Ir(ppy)_3_ was purchased from Luminescence Technology Corp. (Lumtec). NaOH, NaBH_4_, K_2_CO_3_, and anhydrous ethanol were purchased from SAMCHUN Chemicals (Republic of Korea). Benzothien-2-yl boronic acid was purchased from Acros. 2-bromo-4-pyridinecarboxyaldehyde, 2-(4-Fluorophenyl)-4,6-diphenyl-1,3,5-triazine, and 3,6-dibromo-9*H*-carbazole were purchased from Tokyo Chemical Industry Corp. (TCI). 4-vinylbenzyl chloride, sodium hydride (NaH), CF_3_CO_2_Ag, cesium carbonate (Cs_2_CO_3_), 4-vinylphenylboronic acid, anhydrous DMSO, anhydrous *N*,*N*-dimethylformamide (DMF), and anhydrous toluene were purchased from Sigma Aldrich. IrCl_3_∙3H_2_O and Pd(PPh_3_)_4_ were purchased from KM Chemical (Republic of Korea). Phosphoryl chloride (POCl_3_) and the rest of other solvents were purchased from DAEJUNG Chemicals & Metals (Republic of Korea) and used without further purification. Anhydrous toluene and deionized water for Suzuki coupling reaction were degassed by three freeze-pump-thaw cycles.

### Materials for device fabrication

Process solvents, including chloroform (CF), chlorobenzene (CB), acetone, and isopropyl alcohol, were purchased from Sigma-Aldrich. PRs were purchased from Kemlab (KL5301) and AZ Electronic Materials (AZ-5214E). Photoresist developer (AZ 300MIF) was purchased from Merck Performance Materials. Glass substrates with ITO pattern were purchased from AMG (Republic of Korea). PEDOT:PSS (Al4038) was purchased from Ossila, and QUPD was purchased from Lumtec. CBP-V2 (DV-CBP) was purchased from Lumtec. Thermal initiator, 2,2′-Azobis(2-methylpropionitrile) (AIBN), was purchased from DAEJUNG Chemicals & Metals. Lithium fluoride (LiF, ≥99.99% powder) source was purchased from Sigma-Aldrich.

### Synthesis of Ir(ppy)_3_-V3

#### Synthesis of Ir(ppy)_3_-CHO3

To anhydrous DMF (36 mL), POCl_3_ (7.32 mL, 78.28 mmol) was added dropwise to the solution at 0 °C. The mixture was stirred at RT for 1 h. Then, Ir(ppy)_3_ (1192 mg, 1.820 mmol) was added. The resulting solution was stirred at 80 °C for 16 h. Afterward, the reaction mixture was cooled to 0 °C, and a 1 M NaOH aqueous solution (110 mL) was added dropwise. The final reaction mixture was stirred at RT for 6 h. The product was obtained as a yellow solid by filtration and washed with deionized water. The filtered solution was extracted with ethyl acetate (100 mL) and deionized water (100 mL). The combined organic layers were dried over anhydrous MgSO_4_ and concentrated under reduced pressure. The yellow solid was obtained by precipitation with ethyl acetate and *n*-hexane. The resulting yellow solid was combined and used directly in the next step without further purification due to the low stability of the product (1153 mg, 85%). ^1^H-NMR (400 MHz, CDCl_3_) δ: 9.88 (s, 3H), 8.19 (s, 3H), 8.09 (d, J = 8 Hz, 3H), 7.76 (t, J = 8 Hz, 3H), 7.52 (d, J = 4 Hz, 3H), 7.04 (t, J = 8 Hz, 3H), 6.98 (d, J = 8 Hz, 3H).

#### Synthesis of Ir(ppy)_3_-MOH3

To a mixture of Ir(ppy)_3_-CHO3 (1153 mg, 1.561 mmol) and anhydrous ethanol (110 mL), NaBH_4_ (1770 mg, 46.83 mmol) was added. The reaction mixture was stirred at RT for 24 h. The yellow solid was obtained through filtration and washed with deionized water. The product was dried under vacuum and used directly in the next step without further purification due to the low stability of the product (1005 mg, 86%). ^1^H-NMR (400 MHz, DMSO-*d*_6_) δ: 8.09 (d, J = 8 Hz, 3H), 7.78 (t, J = 8 Hz, 3H), 7.69 (s, 3H), 7.46 (d, J = 4 Hz, 3H), 7.10 (t, J = 8 Hz, 3H), 6.65 (q, J = 8 Hz, 3H), 4.87 (s, 3H), 4.35 (s, 6H).

#### Synthesis of Ir(ppy)_3_-V3

To a solution of Ir(ppy)_3_-MOH3 (1005 mg, 1.349 mmol) in anhydrous DMF (20 mL), NaH (809 mg, 20.24 mmol) was added at 0 °C and stirred for 1 h at 0 °C. After 1 h, a solution of 4-vinylbenzyl chloride (2059 mg, 12.14 mmol) in anhydrous DMF (10 mL) was added dropwise to the reaction mixture at 0 °C. The resulting solution was stirred for 24 h at RT. Afterward, deionized water (30 mL) was added to the reaction mixture at 0 °C to quench the reaction. The reaction mixture was extracted three times with dichloromethane (50 mL) and deionized water (50 mL). The combined organic layer was dried using anhydrous MgSO_4_ and concentrated under reduced pressure. The crude product was purified by silica gel column chromatography (dichloromethane: *n*-hexane = 1:1 vol. to only dichloromethane). Finally, the resulting product was precipitated with ethyl acetate and *n*-hexane. The yellow solid was obtained (664 mg, 45%). ^1^H-NMR (400 MHz, CDCl_3_) δ: 7.88 (d, J = 12 Hz, 3H), 7.64 (s, 3H), 7.58 (t, J = 6 Hz, 3H), 7.50 (d, J = 4 Hz, 3H), 7.38 (d, J = 8 Hz, 6H), 7.32 (d, J = 8 Hz, 6H), 6.86‒6.79 (m, 9H), 6.71 (dd, J = 4 Hz, J = 8 Hz, 3H), 5.73 (d, J = 16 Hz, 3H), 5.22 (d, J = 8 Hz, 3H), 4.54 (s, 6H), 4.44 (s, 6H). ^13^C-NMR (100 MHz, CDCl_3_) δ: 166.60, 160.96, 147.24, 144.01, 138.48, 137.17, 136.93, 136.78, 136.09, 130.50, 128.24, 126.34, 124.16, 122.11, 119.05, 113.74, 73.12, 71.71. ESI-MS m/z Calcd: 1093.38 Found: 1093.3792.

### Synthesis of Ir(btpy)_3_-V3

#### Synthesis of 2-(benzo[b]thiophen-2-yl)isonicotinaldehyde (Compound 3)

2-Bromo-4-pyridinecarboxyaldehyde (5.24 g, 25.28 mmol), benzothien-2-yl boronic acid (5.484 g, 30.19 mmol), and Pd(PPh_3_)_4_ were dissolved in anhydrous toluene (58 mL). Anhydrous methanol (38 mL) and a 2 M K_2_CO_3_ aqueous solution (40.75 mL) were added to the mixture. The resulting solution was stirred for 3 h at 105 °C. After 3 h, the reaction mixture was cooled to RT and then extracted three times with dichloromethane (100 mL) and deionized water (100 mL). The combined organic layer was dried using anhydrous MgSO_4_ and concentrated under reduced pressure. The crude product was purified by silica gel column chromatography using dichloromethane as the eluent. The product was recrystallized with dichloromethane and methanol. A white solid was obtained (5.745 g, 85%). ^1^H-NMR (400 MHz, CDCl_3_) δ: 10.14 (s, 1H), 8.87 (d, J = 4 Hz, 1H), 8.18 (s, 1H), 7.96 (s, 1H), 7.90‒7.83 (m, 2H), 7.60 (d, J = 4 Hz, 1H), 7.41‒7.37 (m, 2H). ^13^C-NMR (100 MHz, CDCl_3_) δ: 191.40, 154.37, 151.26, 143.66, 142.35, 141.02, 140.40, 125.72, 124.89, 124.57, 122.79, 122.50, 121.04, 118.06. ESI-MS m/z Calcd: 239.04 Found: 240.0476 (M + H).

#### Synthesis of (2-(benzo[b]thiophen-2-yl)pyridin-4-yl)methanol (Compound 4)

To a solution of 2-(benzo[*b*]thiophen-2-yl)isonicotinaldehyde (5.745 g, 24.01 mmol) in anhydrous ethanol (200 mL), NaBH_4_ (1.362 g, 36.01 mmol) was added at RT. The reaction mixture was stirred at RT for 1 h. Subsequently, the reaction mixture was extracted three times with ethyl acetate (150 mL) and deionized water (150 mL). The combined organic layer was dried using anhydrous MgSO_4_ and concentrated under reduced pressure. The product was precipitated with *n*-hexane. A white solid was obtained (5.3 g, 91%). ^1^H-NMR (400 MHz, CDCl_3_) δ: 8.58 (d, J = 4 Hz, 1H), 7.88‒7.79 (m, 4H), 7.38‒7.33 (m, 2H), 7.18 (d, J = 4 Hz, 1H), 4.81 (d, J = 8 Hz, 2H), 2.02 (t, J = 4 Hz, 1H). ^13^C-NMR (100 MHz, CDCl_3_) δ: 152.81, 150.85, 149.75, 144.71, 140.73, 140.54, 125.23, 124.66, 124.27, 122.71, 121.44, 120.21, 117.00, 63.61. ESI-MS m/z Calcd: 241.06 Found: 242.0635 (M + H).

#### Synthesis of 2-(benzo[b]thiophen-2-yl)-4-(((4-vinylbenzyl)oxy)methyl)pyridine (Compound 5)

To a solution of (2-(benzo[*b*]thiophen-2-yl)pyridin-4-yl)methanol (5300 mg, 21.96 mmol) in anhydrous DMF (50 mL), NaH (1317 mg, 32.95 mmol) was slowly added at 0 °C and stirred for 1 h at 0 °C. After 1 h, a solution of 4-vinylbenzyl chloride (7434 mg, 43.92 mmol) in anhydrous DMF (20 mL) was added dropwise to the reaction mixture at 0 °C. The resulting solution was stirred for 2 h at RT. Afterward, deionized water (70 mL) was added to the reaction mixture at 0 °C to quench the reaction. The reaction mixture was extracted three times with dichloromethane (50 mL) and deionized water (50 mL). The combined organic layer was dried using anhydrous MgSO_4_ and concentrated under reduced pressure. The crude product was purified by silica gel column chromatography using dichloromethane as the eluent. Finally, the resulting product was recrystallized with dichloromethane and methanol. The white solid was obtained (6709 mg, 85%). ^1^H-NMR (400 MHz, CDCl_3_) δ: 8.59 (d, J = 8 Hz, 1H), 7.88‒7.79 (m, 4H), 7.44 (d, J = 8 Hz, 2H), 7.37‒7.34 (m, 4H), 7.18 (d, J = 4 Hz, 1H), 6.74 (dd, J = 4 Hz, J = 8 Hz, 1H), 5.78 (d, J = 20 Hz, 1H), 5.26 (d, J = 12 Hz, 1H), 4.64 (s, 2H), 4.61 (s, 2H). ^13^C-NMR (100 MHz, CDCl_3_) δ: 152.85, 149.84, 148.41, 144.91, 140.80, 140.59, 137.51, 137.23, 136.57, 136.55, 128.23, 126.55, 125.18, 124.63, 124.25, 122.72, 121.35, 120.97, 117.71, 114.27, 72.74, 70.39. ESI-MS m/z Calcd: 357.12 Found: 358.1257 (M + H).

#### Synthesis of Ir dimer

To a solution of IrCl_3_∙3H_2_O (450 mg, 1.276 mmol) in 2-ethoxyethanol (30 mL) and deionized water (10 mL), 2-(benzo[*b*]thiophen-2-yl)-4-(((4-vinylbenzyl)oxy)methyl)pyridine (1003 mg, 2.808 mmol) was added. The resulting solution was stirred for 24 h at 120 °C. After stirring, the reaction mixture was cooled to RT. The red solid was obtained through filtration and washed with deionized water and methanol. The product was dried under vacuum and used directly in the next step without further purification (1044 mg, 87%).

#### Synthesis of Ir(btpy)_3_-V3

To a solution of Ir dimer (1044 mg, 0.555 mmol) and 2-(benzo[*b*]thiophen-2-yl)-4-(((4-vinylbenzyl)oxy)methyl)pyridine (794 mg, 2.220 mmol) in 2-ethoxyethanol (40 mL), CF_3_CO_2_Ag (368 mg, 1.665 mmol) was added. The resulting solution was stirred for 24 h at 120 °C. After stirring, the reaction mixture was cooled to RT and extracted three times with dichloromethane (60 mL) and deionized water (60 mL). The combined organic layer was dried using anhydrous MgSO_4_ and concentrated under reduced pressure. The crude product was purified by silica gel column chromatography using dichloromethane as the eluent. Finally, the resulting product was precipitated with dichloromethane and methanol. The red solid was obtained (166 mg, 12%). ^1^H-NMR (400 MHz, CDCl_3_) δ: 7.94 (d, J = 8 Hz, 1H), 7.78 (m, 3H), 7.60 (d, J = 4 Hz, 2H), 7.58 (s, 1H), 7.51 (s, 1H), 7.42 (s, 1H), 7.41‒7.39 (m, 6H), 7.34‒7.32 (m, 6H), 7.13‒7.06 (m, 3H), 6.82‒6.71 (m, 7H), 6.63‒6.54 (m, 3H), 6.24 (t, J = 8 Hz, 2H), 5.76 (d, J = 20 Hz, 3H), 5.27‒5.23 (m, 3H), 4.63‒4.58 (m, 12H). ^13^C-NMR (100 MHz, CDCl_3_) δ: 176.13, 172.11, 166.61, 165.06, 163.94, 154.88, 154.49, 151.54, 149.80, 149.04, 148.39, 148.35, 147.84, 147.32, 144.37, 143.97, 142.76, 138.92, 137.93, 137.53, 137.41, 137.21, 137.03, 137.01, 136.54, 132.43, 128.77, 128.30, 128.23, 126.56, 126.52, 125.07, 124.96, 123.81, 123.57, 122.89, 122.76, 122.12, 119.16, 118.36, 116.89, 116.59, 116.89, 116.59, 116.05, 114.27, 114.16, 73.06, 72.75, 72.51, 70.11, 69.98. ESI-MS m/z Calcd: 1261.296 Found: 1261.2959.

### Synthesis of TRTz-V2

#### Synthesis of 3,6-bis(4- styryl)-9H-carbazole (Compound 6)

3,6-Dibromo-9*H*-carbazole (1.000 g, 3.077 mmol) and (4-vinylphenyl)boronic acid (1.130 g, 7.639 mmol) were dissolved in 60 mL of toluene in an oven-dried 250 mL round-bottom flask equipped with a condenser and a magnetic stir bar. Water (20 mL) and methanol (1:1, v/v) were added to the reaction mixture. The resulting solution was purged with a stream of Ar gas for 20 min. After that, Na_2_CO_3_ (1.300 g, 12.265 mmol) and Pd(PPh_3_)_4_ (0.900 g, 0.779 mmol) were subsequently added to the solution. The reaction mixture was heated at 85 °C for 12 h under an Ar atmosphere. After cooling the reaction mixture to room temperature, the solvent was evaporated under reduced pressure. The concentrate was dissolved in CH_2_Cl_2_ and filtered through a Celite pad to remove solid particles. The filtrate was poured onto water, and the crude product was extracted with CH_2_Cl_2_ (100 mL, three times). The combined organic layers were dried over anhydrous MgSO_4_, filtered, and concentrated under reduced pressure. Finally, silica gel column chromatography was performed using an EtOAc:*n*-hexane (1:9, v/v) as the eluent to afford 0.800 g of white solid (70% yield). ^1^H NMR (300 MHz, DMSO-*d*_6_) δ: 11.41 (s, 1H), 8.63 (s, 2H), 7.81 (d, *J* = 8.1 Hz, 4H), 7.77 (dd, *J* = 8.4, 1.8 Hz, 2H), 7.56‒7.60 (m, 6H), 6.80 (dd, *J* = 17.7, 11.1 Hz, 2H), 5.89 (dd, *J* = 17.7, 0.6 Hz, 2H), 5.29 (dd, *J* = 11.1, 0.9 Hz, 2H). ^13^C NMR (150 MHz, CDCl_3_): *δ* 141.6, 139.6, 136.7, 132.9, 127.5, 126.9, 125.7, 124.2, 118.9, 113.7, 111.2.

#### Synthesis of TRTz-V2

3,6-Bis(4-styryl)-9*H*-carbazole (0.100 g, 0.269 mmol) and Cs_2_CO_3_ (0.130 g, 0.399 mmol) were suspended in 8 mL of DMSO in an oven-dried 25 mL round-bottom flask equipped with a condenser and a magnetic stir bar. The resulting mixture was stirred for 15 min at room temperature. 2-(4-Fluorophenyl)-4,6-diphenyl-1,3,5-triazine (0.088 g, 0.269 mmol) was added to the reaction mixture, which was heated at 110 °C for 10 h. The depletion of starting material is monitored by TLC. The reaction mixture was cooled to room temperature and poured onto ice water. The resultant yellow precipitate was washed with ethanol several times to afford 0.111 g of a yellow solid (62%). ^1^H NMR (300 MHz, CDCl_3_) *δ*: 9.08 (d, *J* = 8.6 Hz, 2H), 8.86 (dd, *J* = 7.6, 1.4 Hz, 4H), 8.46 (s, 2H), 7.89 (d, *J* = 8.6 Hz, 2H), 7.76 (dd, *J* = 8.6, 1.4 Hz, 6H), 7.61−7.71 (m, 8H), 7.56 (d, *J* = 8.2 Hz, 4H), 6.82 (dd, *J* = 17.6, 10.9 Hz, 2H), 5.85 (d, *J* = 17.6 Hz, 2H), 5.31 (d, *J* = 10.9 Hz, 2H). ^13^C NMR (150 MHz, CDCl_3_): *δ* 172.0, 171.1, 141.6, 141.3, 140.6, 136.7, 136.3, 135.3, 133.8, 132.9, 130.9, 129.2, 129.1, 128.9, 127.5, 126.9, 126.7, 125.9, 124.6, 119.0, 113.8, 110.6.

### Patterning processes

Initially, KL 5301 (PR) was spin-coated onto the substrate at 4500 rpm for 40 s. Subsequently, it was exposed to a UV source (i-line, 9.8 mW/cm^2^) for 9 s using a photomask and a mask aligner (MIDAS system, MDA-400LJ). The UV exposed PR was developed using the AZ 300 MIF developer. Following this, organic luminophores (6 mg mL^−1^ in chloroform or 14 mg mL^−1^ in chlorobenzene) were spin-coated (2000 rpm for 60 s) onto the PR pattern. For R/G/B EMLs, we used Ir(ppy)3-V3 (12 wt%) for green emission, Ir(btpy)3-V3 (12 wt%) for red emission, and TRTz-V2 (10 wt%) for blue emission, along with CBP-V2 host and the thermal initiator (AIBN, 1 wt%). Next, the EML was subjected to thermal annealing (110 °C, 10 min). Subsequently, the substrate with the crosslinked EML was soaked in the stripping solvent (acetone or acetonitrile) for 30 min. Finally, the PR pattern on the resulting substrate was removed through a sonication process for 1 min, thereby creating micropatterns of OLEDs EML. If complete PR removal was not achieved, additional sonication steps were performed as needed. We note that the selection of the stripping solvent was critical in minimizing damage to the EML during this step. For instance, DMSO, which is conventionally used as a stripping solvent for PR (KL5301 or AZ-5214E), was found to cause damage to the crosslinked EML pattern during the sonication process. Instead, we found that acetone or acetonitrile is more suitable as a benign stripping solvent.

### Device fabrication

The ITO patterned substrates underwent a sequential cleaning process involving acetone, isopropyl alcohol, and distilled water. For OLED fabrication, PEDOT:PSS (Al4083, 4500 rpm for 60 s) was spin-coated onto the ITO substrate, followed by thermal annealing (110 °C, 10 min) in the glove box. QUPD HTL (10 mgmL^−1^ in chlorobenzene) was then spin-coated (2000 rpm for 60 s) onto the PEDOT:PSS layer, followed by thermal annealing (180 °C, 30 min) for the polymerization of oxetane groups. Subsequently, the EML was formed. Specifically, organic luminophores added with AIBN (6 mg mL^−1^ in chloroform or 14 mg mL^−1^ in chlorobenzene) were directly spin-coated (2000 rpm for 60 s) onto the crosslinked QUPD layer without the pre-formed sacrificial PR pattern. For R/G/B EMLs, we used Ir(ppy)3-V3 (12 wt%) for green emission, Ir(btpy)3-V3 (12 wt%) for red emission, and TRTz-V2 (10 wt%) for blue emission, along with CBP-V2 host and the thermal initiator (AIBN, 1 wt%). The spin-coated film was thermally annealed (110 °C, 10 min) to induce crosslinking, and the resulting crosslinked film was rinsed with acetone or acetonitrile to mimic the stripping condition in the patterning process. Details of the optimization process for determining the doping ratio in OLEDs are provided in Fig. S17. When fabricating the devices with patterned EML (that includes all the patterning processes described above), we noticed that the underlying PEDOT:PSS layer was swollen by the stripping solvent used in the last step of the patterning process. If swelling happened significantly, sometimes the entire EML layer was removed completely during the stripping step. Consequently, the stripping step had to be done delicately in a way to minimize the swelling of the underlying PEDOT:PSS layer. An alternative way to safely leave the EML pattern was to selectively etch the EML layer that is placed on top of the sacrificial PR pattern, so that the sacrificial PR pattern is exposed. To enable selective etching, we employed a second PR (AZ-5214E) selectively formed onto the emissive region of the EML. This second PR pattern served as a protective layer during reactive-ion etching. Once the reactive ion etching was performed using Ar/O_2_ (40 sccm/20 sccm) etching gases for 20 s with 50 W radio-frequency power under a pressure of 0.05 torr), the underlying sacrificial PR pattern as well as the second PR pattern were removed through a sonication process using acetone and isopropyl alcohol, resulting in the EML pattern formed onto the QUPD layer. Subsequently, TPBi ETL was thermally evaporated onto the EML at a deposition rate of 10 Å s^−1^. Finally, the LiF:Al electrode was thermally evaporated onto the TPBi layer at a deposition rate each 1 Å s^−1^ for LiF, 30 Å s^−1^ for Al.

### Measurement and characterization

To identify the molecular structures of all the synthesized products, ^1^H-NMR and ^13^C-NMR spectra were measured by Agilent 400 MHz FT-NMR. Mass spectra of synthetic materials were obtained by electrospray ionization-mass spectrometry (ESI) technique using AccuTOF 4 G + DART (JEOL, Japan). To characterize the crosslinking reaction of the EML, FT-IR spectra were measured using an FT-IR4700 (Jasco) in an attenuated total reflection (ATR) mode using bare Si substrates. The surface morphology and the height profile of the EML and pattern were measured using a Park XE7 AFM system (Park Systems). UV-Vis spectra were measured using a V-770 UV-visible/NIR spectrophotometer (Jasco). PL spectra were measured using an FS-2 fluorescence spectrometer (Scinco). Transient photoluminescence (Transient PL) was conducted using a time-correlated single photon counting (TCSPC) system. Absolute photoluminescence quantum yield (PLQY) was measured by a Quantaurus-QY absolute PL quantum yield spectrometer (Hamamatsu Photonics). Fluorescence images of the EML pattern were captured using optical microscopy under 365 nm UV light irradiation. Cyclic voltammograms of the organic luminophores were attained using a VSP-300 (BioLogic) using a 2:1 mixture of chlorobenzene and acetonitrile (10^−4^ M) as the solvent and tetra-n-butylammonium hexafluorophosphate (TBAPF_6_) as the supporting electrolyte. A ferrocenium/ferrocene (Fc/Fc^+^) couple was employed as the external reference. The EL characteristics of the OLEDs were evaluated by a PR 655 spectroradiometer (JADAK) while the input voltage was swept from 0 V to 13 V using a Keithley 2400.

## Supplementary information

Supplementary Information

## Data Availability

All data are available in the manuscript or the supplementary information.
