# Supplementary material for: Micrometer-scale indirect photopatterning of RGB OLED emissive layers in single phase network structure
Source: Light Sci Appl. 2025 Jul 22;14:247. doi: 10.1038/s41377-025-01907-w (PMC12284257; doi:10.1038/s41377-025-01907-w)
Supplement: Supplementary file 1 — Supplementary Information [file 41377_2025_1907_MOESM1_ESM.docx]

Supplementary Information for

Micrometer-scale Indirect Photopatterning of RGB OLED Emissive Layers in Single Phase Network Structure

Seunghan Lee^1^†, Hyobin Ham^2^†, Shahid Ameen^2^, Byung Hak Jhun^3^, SeungHwan Roh^1^, Hyeono Yee^1^, Chang Hyeok Lim^1^, Yuchan Heo^2^, Hyukmin Kweon^4^, Dongheon Han^1^, Do Hwan Kim^4,5^, Youngmin You^3^, BongSoo Kim^2,6^*, and Moon Sung Kang^1,7^*

^1^Department of Chemical and Biomolecular Engineering, Sogang University, Seoul 04107, Republic of Korea

^2^Department of Chemistry, Ulsan National Institute of Science and Technology (UNIST), Ulsan 44919, Republic of Korea

^3^Department of Chemical and Biomolecular Engineering, Yonsei University, Seoul 03722, Republic of Korea

^4^Department of Chemical Engineering, Hanyang University, Seoul 04763, Republic of Korea

^5^Institute of Nano Science and Technology & Clean-Energy Research Institute, Hanyang University, Seoul 04763, Republic of Korea

^6^Graduate School of Semiconductor Materials and Device Engineering & Graduate School of Carbon Neutrality, Ulsan National Institute of Science and Technology (UNIST), Ulsan 44919, Republic of Korea

^7^Institute of Emergent Materials, Sogang University, Seoul 04107, Republic of Korea

†These authors contributed equally to this work.

*Corresponding author. E-mail: [bongsoo@unist.ac.kr](mailto:bongsoo@unist.ac.kr) (B.K.); [kangms@sogang.ac.kr](mailto:kangms@sogang.ac.kr) (M.S.K.)

**Keywords:** organic light-emitting diode (OLED); indirect photopatterning; photoresist; single phase network; vinylbenzyl group


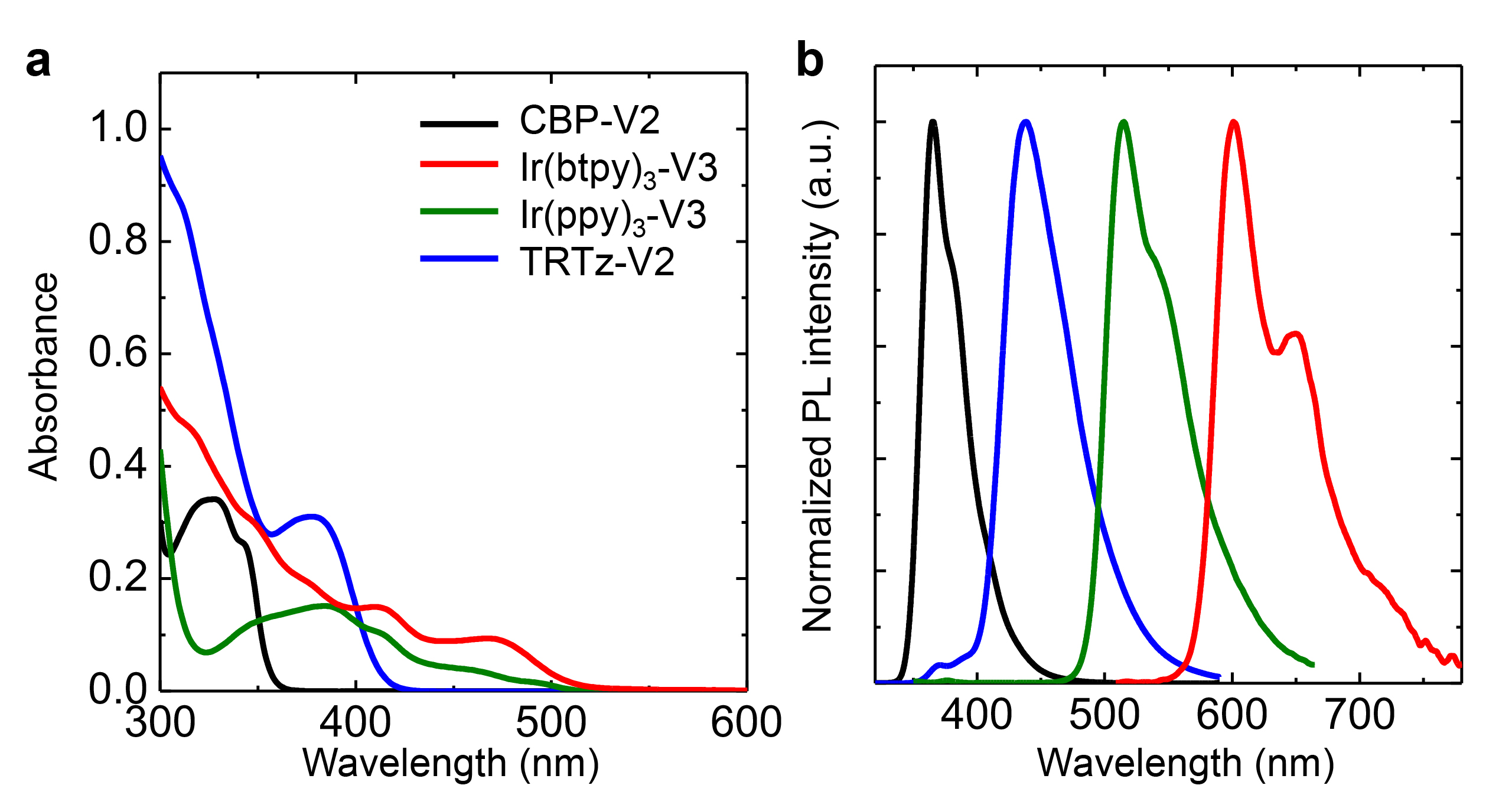


**Supplementary Figure 1 | Absorbance and photoluminescence (PL) spectra of crosslinkable organic luminophore solutions.** **a** The absorbance spectra of organic luminophore solutions in toluene (10^-5^ M). **b** The PL spectra of organic luminophore solutions in toluene (10^-5^ M). For the PL measurements, we used an excitation wavelength of 300 nm for CBP-V2 and TRTz-V2, 340 nm for Ir(ppy)_3_-V3, and 400 nm for Ir(btpy)_3_-V3.

**Supplementary Table 1 |** PL characteristics of crosslinkable organic luminophore solution in toluene (10^-5^ M) under nitrogen atmosphere.

|  | **Absorbance peak**  **(nm)** | **PL peak**  **(nm)** | ***E*_g_**^a^  **(eV)** | **HOMO/LUMO**^b^  **(eV)** | | **PLQY**^c^  **(%)** |
| --- | --- | --- | --- | --- | --- | --- |
| CBP-V2 | 325 | 365 | 3.41 | -5.76/-2.35 | | 81 |
| Ir(btpy)_3_-V3 | 315, 410, 470 | 601 | 1.95 | -5.43/-3.48 | | 51 |
| Ir(ppy)_3_-V3 | 387, 412 | 515 | 2.37 | | -5.44/-3.07 | 28 |
| TRTz-V2 | 377 | 438 | 2.92 | | -5.97/-3.05 | 77 |

^a^The optical bandgap (*E*_g_) estimated from the Tauc plot. ^b^Estimated from the cyclic voltammogram and *E*_g_. ^c^The PL quantum yield (PLQY) was estimated by absolute PL quantum yield spectrometer.


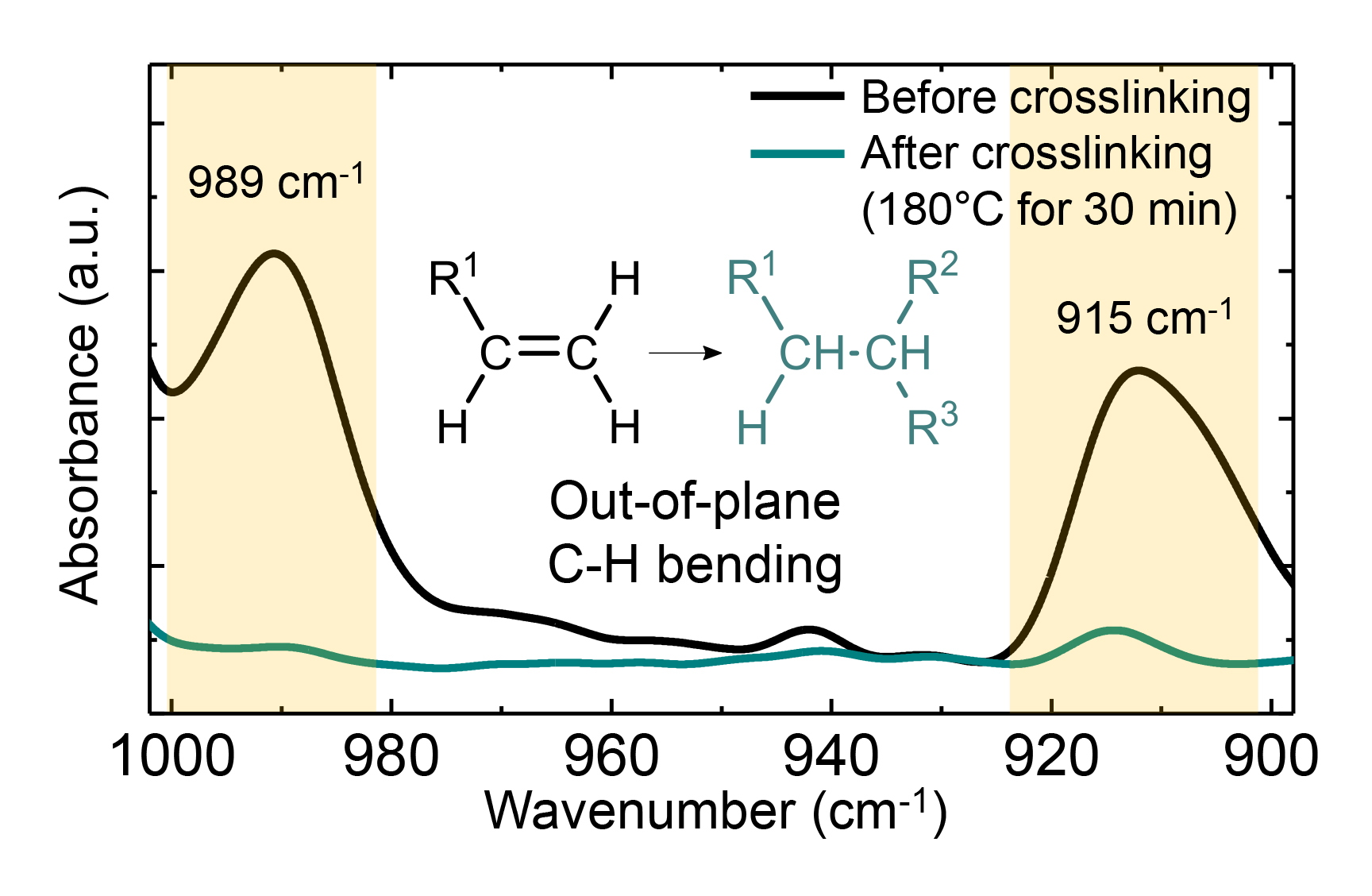


**Supplementary Figure 2 | Evaluation of crosslinking reaction under high temperature annealing.** FT-IR spectra of the green EML film before and after the thermal crosslinking reaction at 180°C for 30 min, indicating the near-complete disappearance of out-of-plane C-H bending mode.


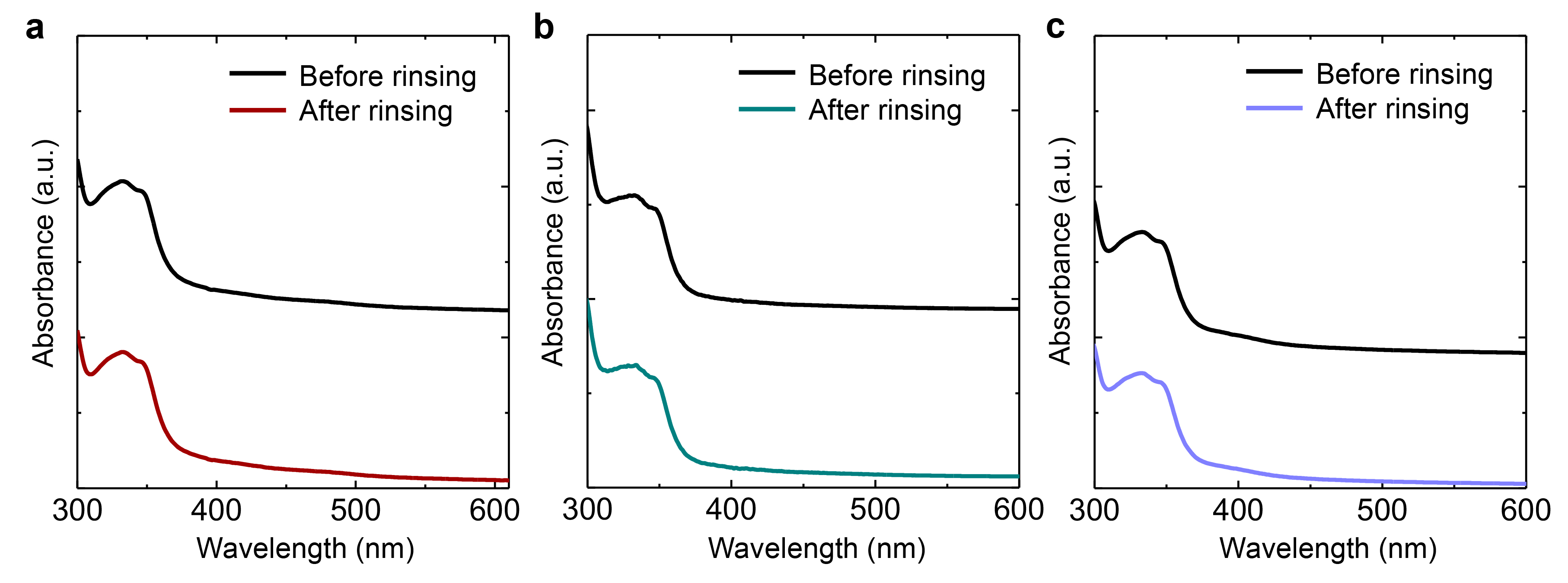


**Supplementary Figure 3 | Evaluation of chemical resistance of crosslinked emissive layer under high temperature annealing.** The absorbance spectra of crosslinked emissive layer film before and after rinsing with the mother solvents. The absorbance spectra of **a** red, **b** green, and **c** blue emissive layer crosslinked at 180°C for 30 minutes.

**
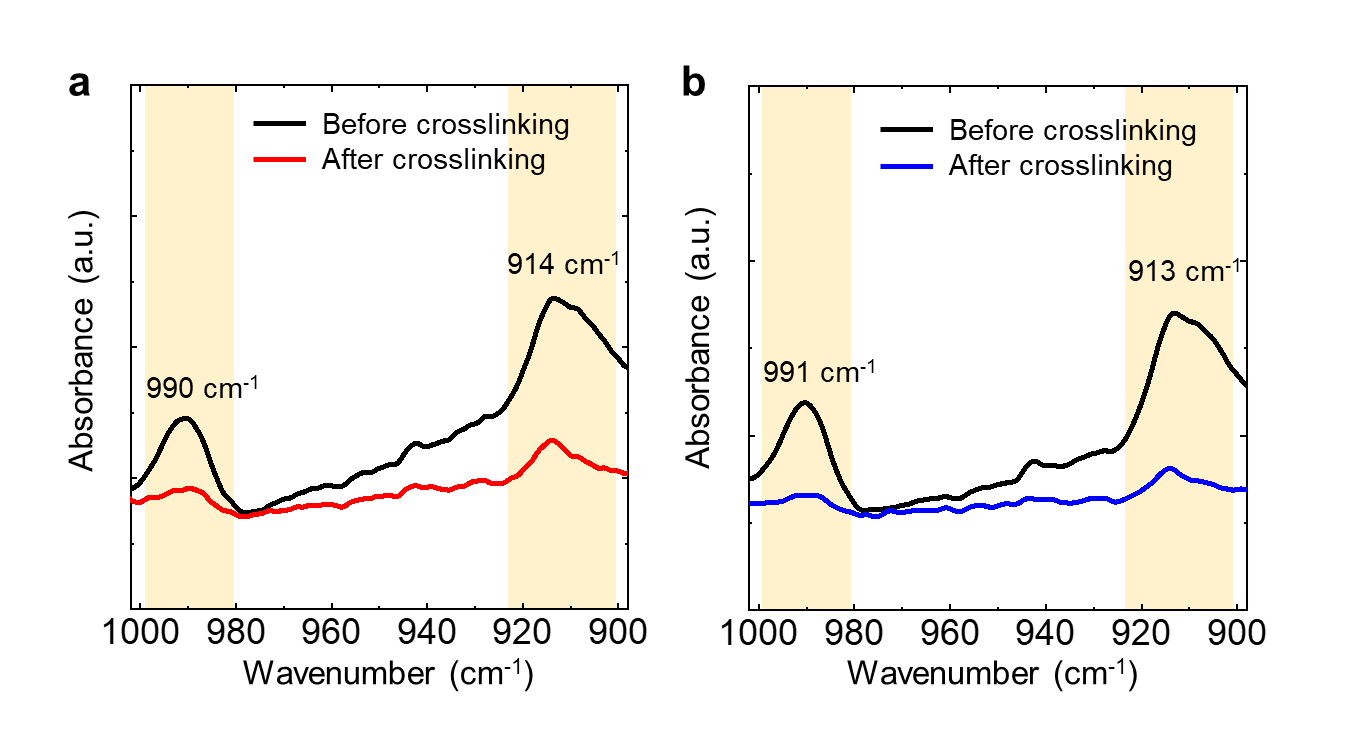
**

**Supplementary Figure 4 | Evaluation of crosslinking reaction in red and blue emissive layer under low temperature annealing.** FT-IR spectra of **a** red and **b** blue EML films before and after the thermal crosslinking reaction at 110°C for 10 min with AIBN, indicating the near-complete disappearance of out-of-plane C-H bending mode.


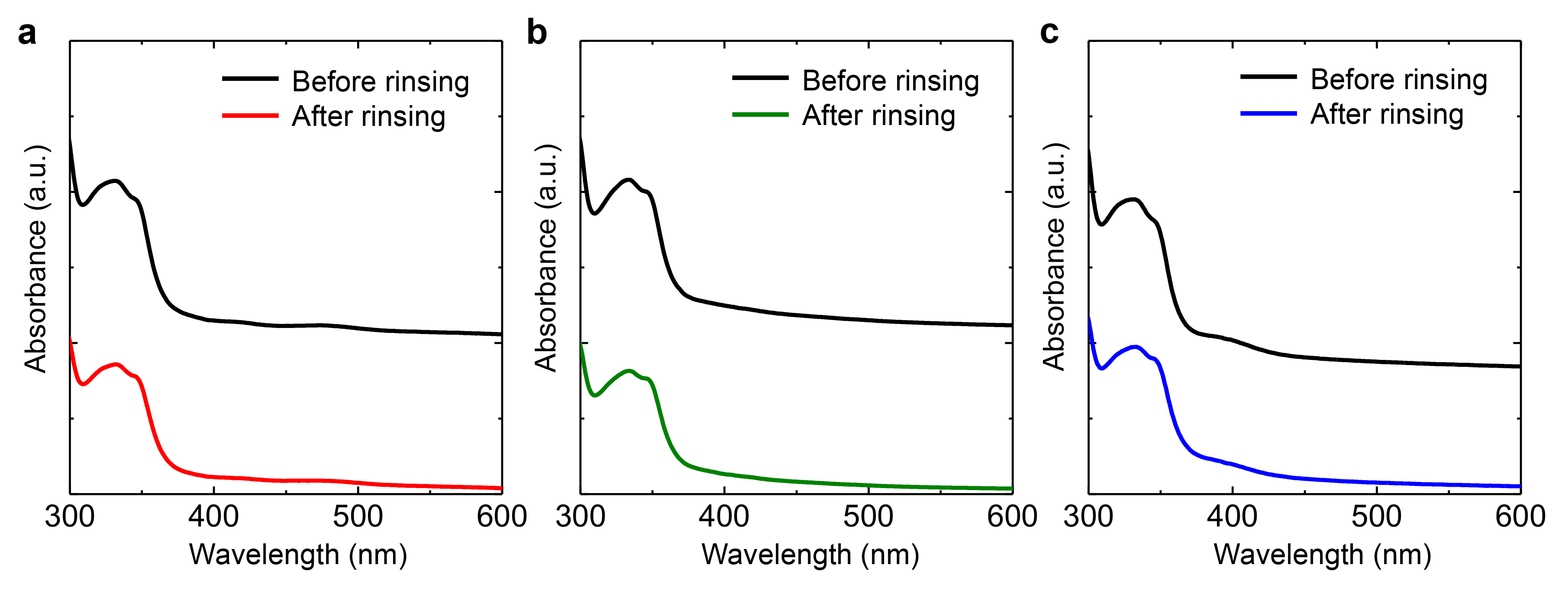


**Supplementary Figure 5 | Evaluation of chemical resistance of crosslinked emissive layer under low temperature annealing.** The absorbance spectra of crosslinked emissive layer film before and after rinsing with the mother solvents. The absorbance spectra of **a** red, **b** green, and **c** blue emissive layer crosslinked at 110°C for 10 minutes with AIBN.


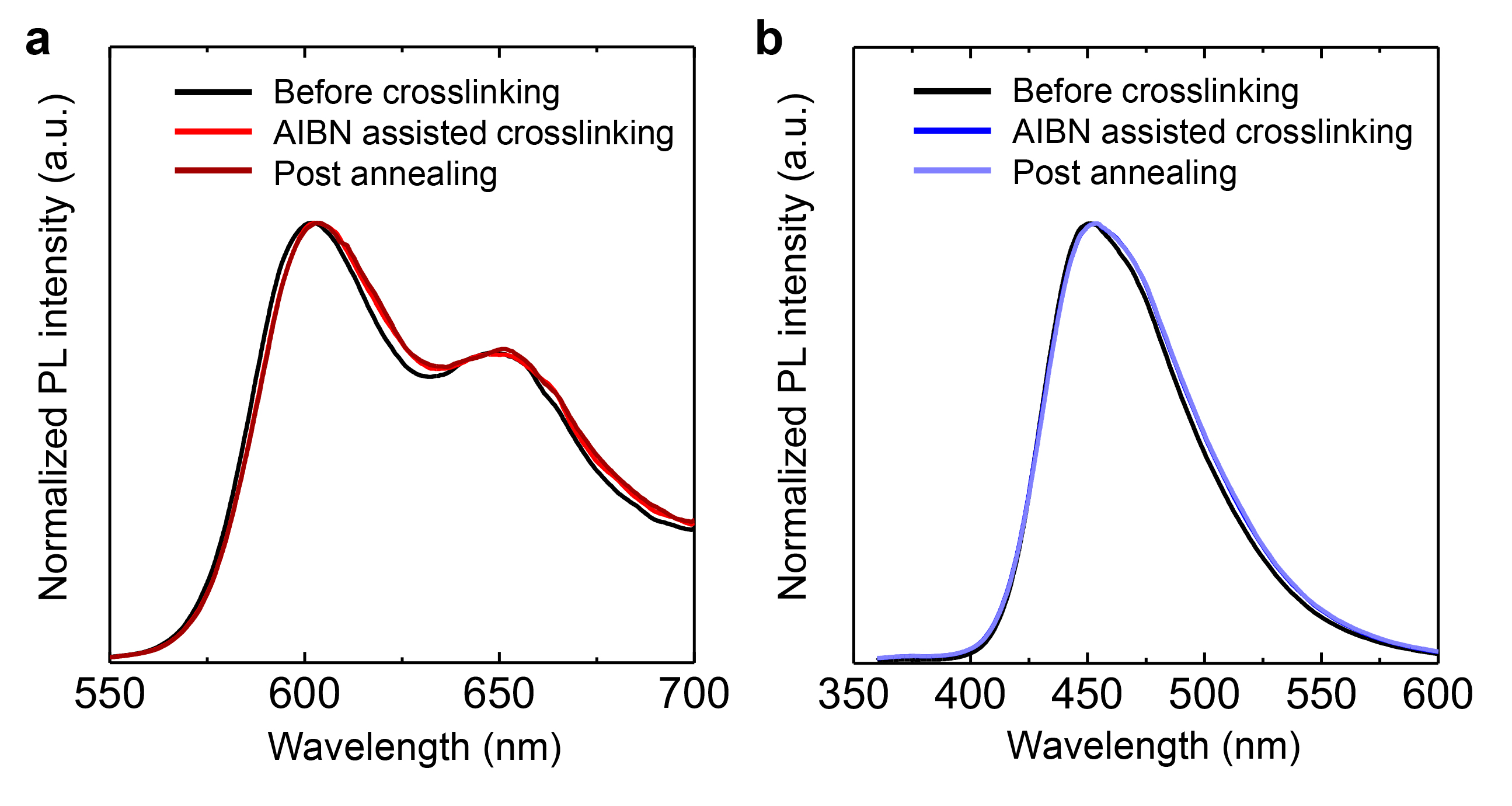


**Supplementary Figure 6 | PL spectra of red/blue emissive layer film under crosslinking conditions.** The SPN structure formation with AIBN involved annealing at 110°C for 10 minutes, and the post annealing condition involved annealing at 180°C for 30 minutes. The PL spectra of **a** red and **b** blue emissive layer.


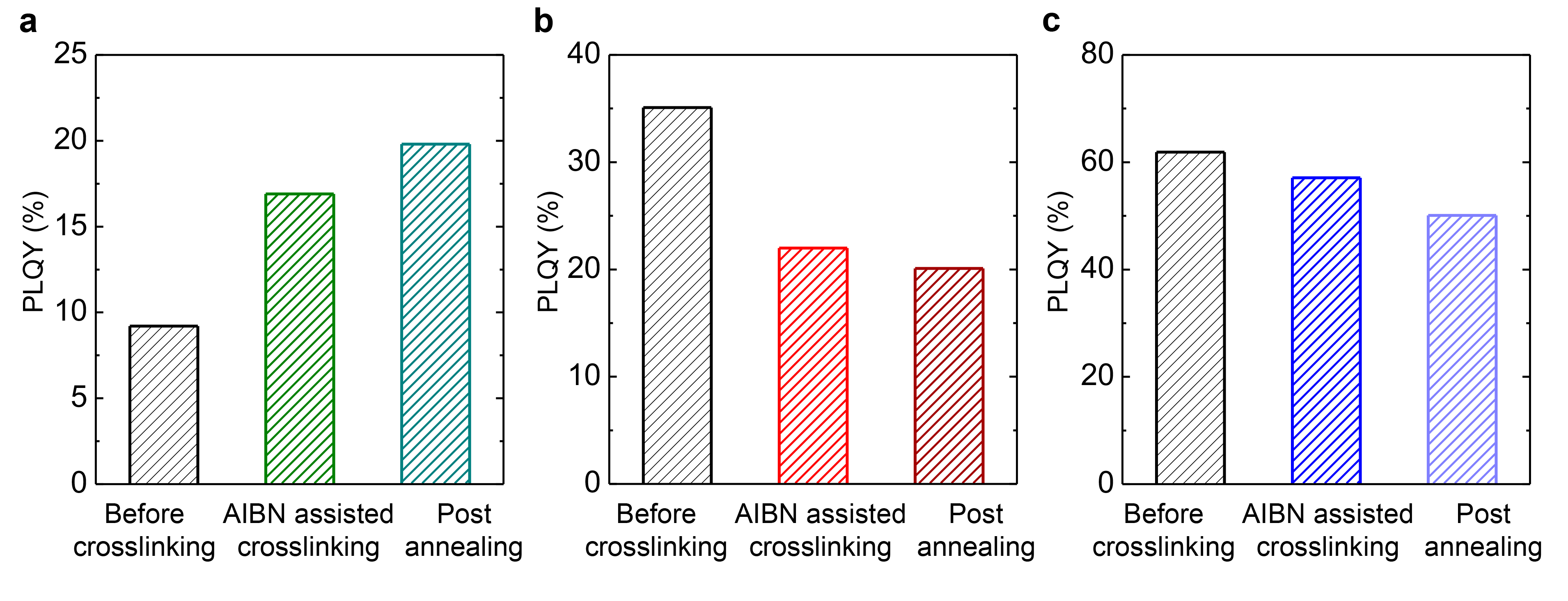


**Supplementary Figure 7 | PLQY of green/red/blue emissive layer film under crosslinking conditions.** The AIBN assisted crosslinking condition involved annealing at 110°C for 10 minutes with AIBN, and the post annealing condition involved annealing at 180°C for 30 minutes. The variations in the PLQY of **a** green, **b** red, and **c** blue emissive layer.

**
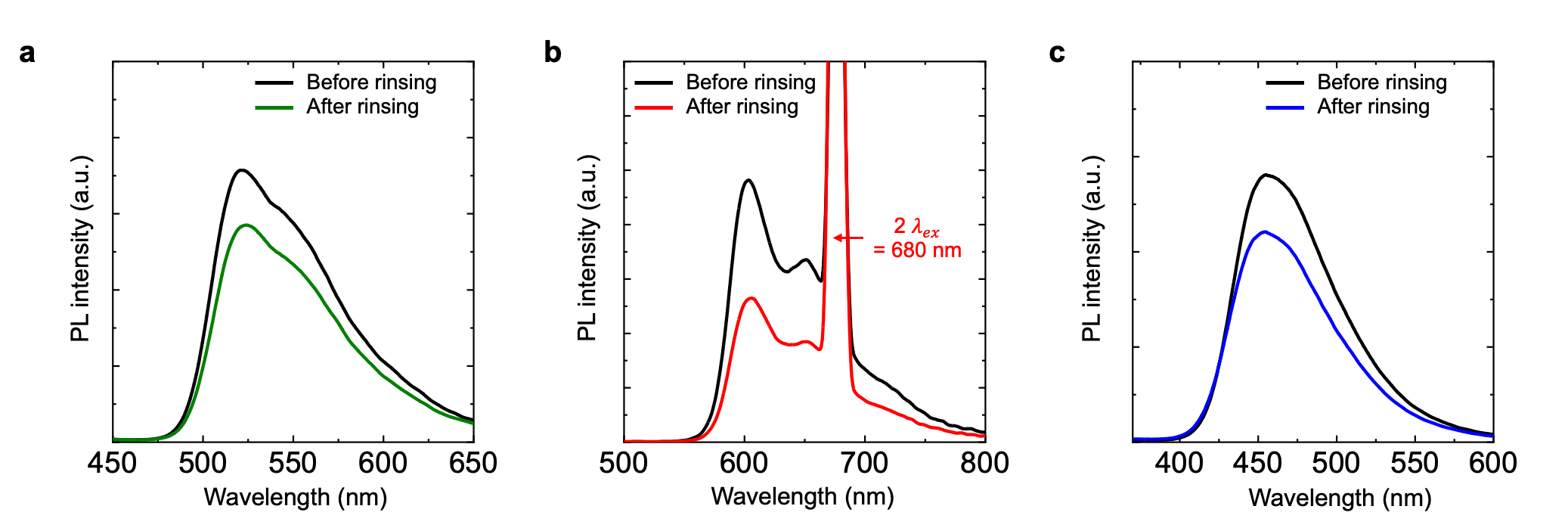
**

**Supplementary Figure 8 | Non-normalized PL spectra of crosslinked green/red/blue emissive layer films before and after rinsing with the mother solvents.** Non-normalized PL spectra of **a** green, **b** red, and **c** blue emissive layer crosslinked at 110°C for 10 minutes with AIBN. The abnormal peak in the red EML PL spectra is double the excitation wavelength (680 nm).

**
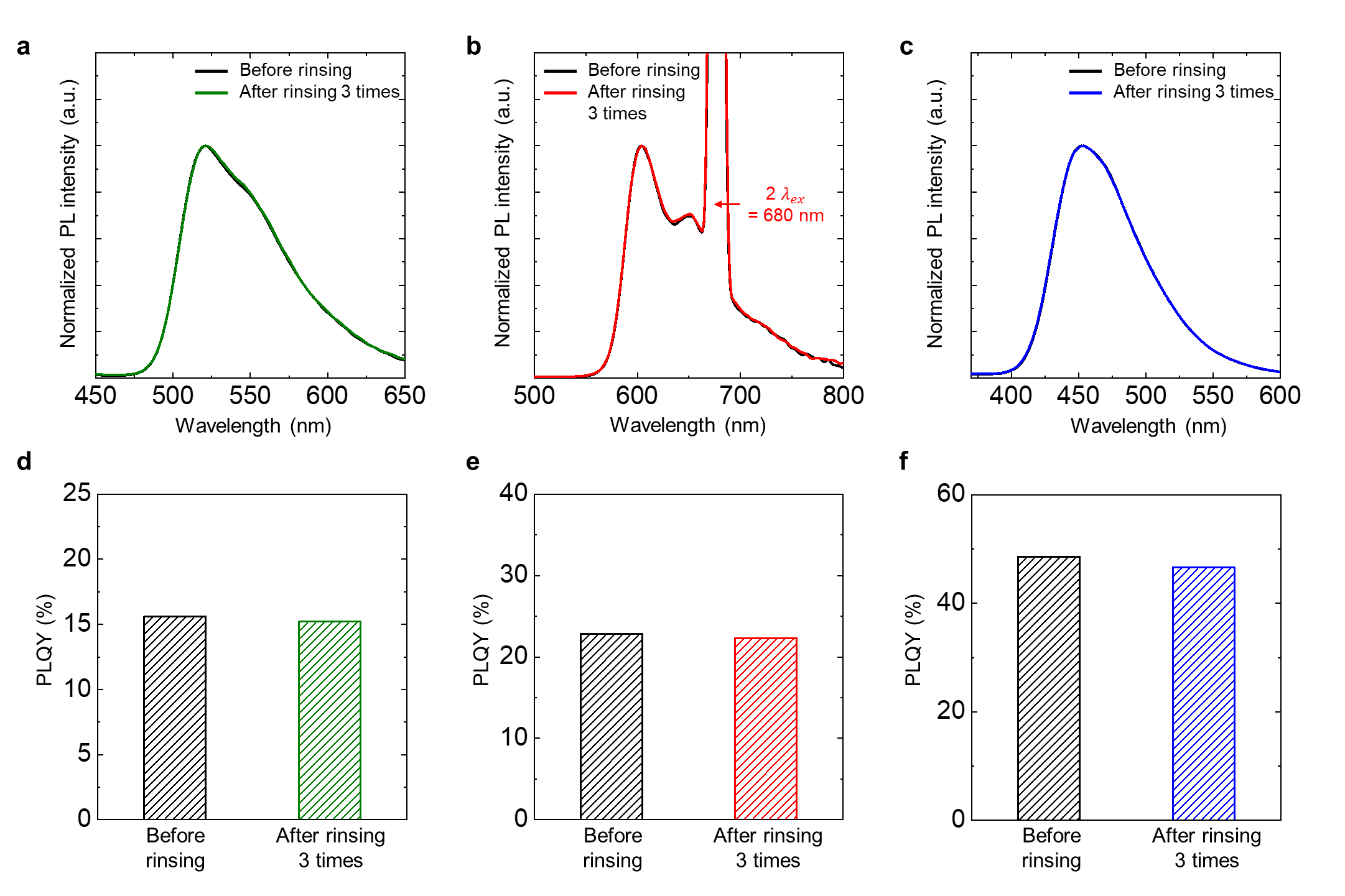
**

**Supplementary Figure 9 | PL properties of crosslinked green/red/blue emissive layer films before and after three rinsing cycles with the mother solvents.** normalized PL spectra of **a** green, **b** red, and **c** blue emissive layer before and after three rinsing cycles. The abnormal peak in the red EML PL spectra is double the excitation wavelength (680 nm). PLQY of **d** green, **e** red, and **f** blue EML films before (blank bar) and after (striped bar) three rinsing cycles.

**
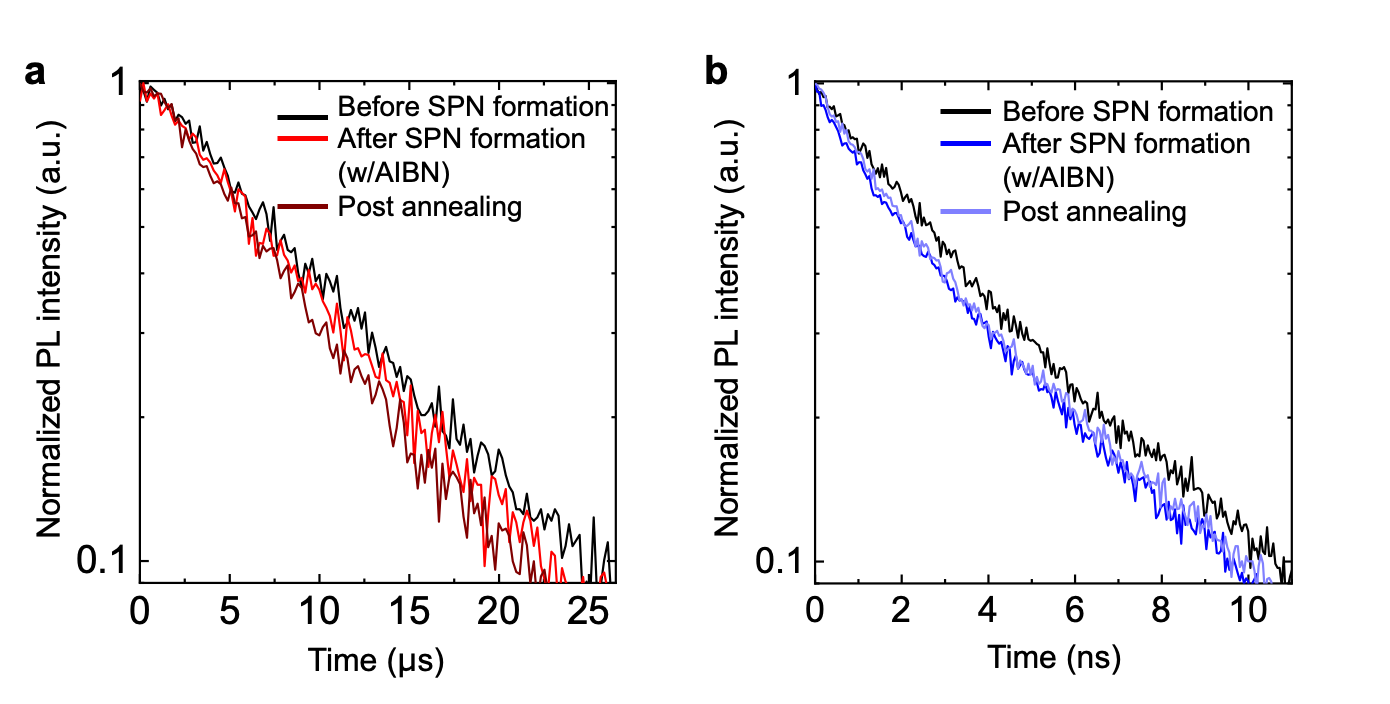
**

**Supplementary Figure 10 | Transient PL spectra of the red/ blue emissive layer films under crosslinking conditions.** The SPN structure formation with AIBN involved annealing at 110°C for 10 minutes, and the post annealing condition involved annealing at 180°C for 30 minutes. The transient PL spectra of **a** red and **b** blue emissive layer.


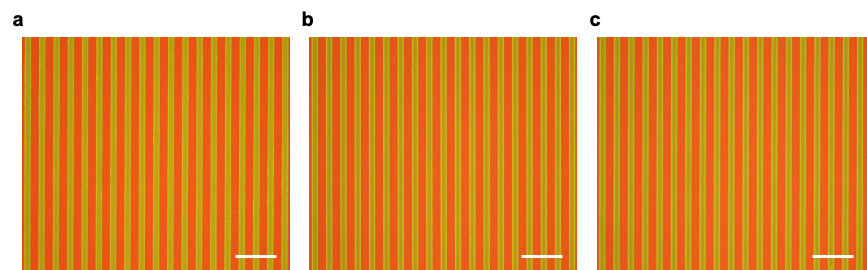


**Supplementary Figure 11 | Red / green / blue (R/G/B) EML patterns without UV radiation**. Large-area OM images of line-shaped (width: 3 µm; spacing: 4 µm) **a** red, **b** green, and **c** blue EML patterns. The scale bar is 20 μm.

**
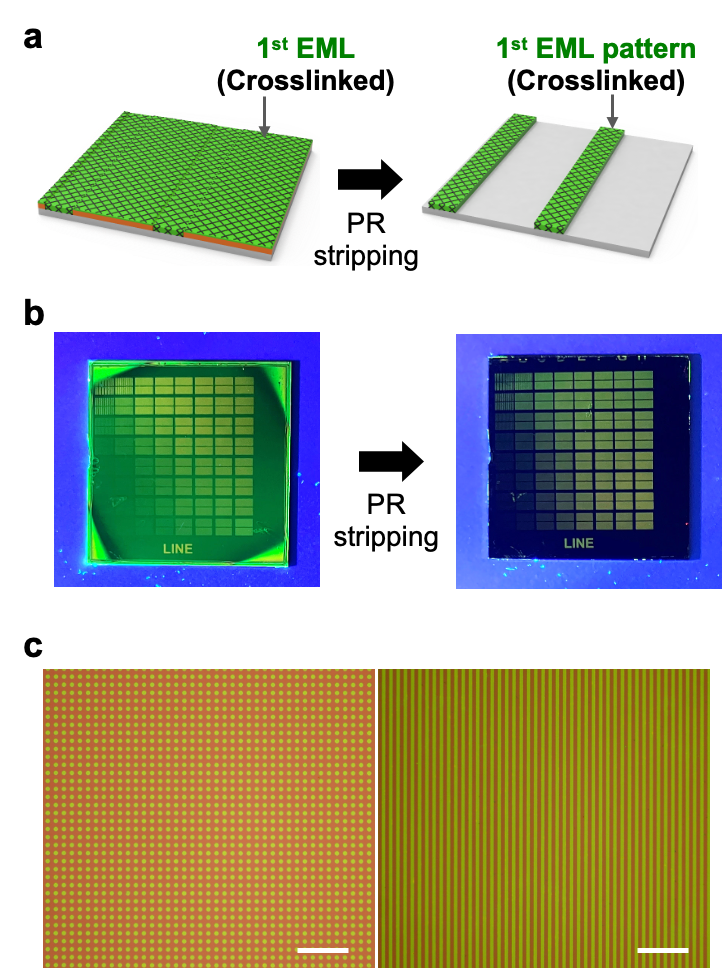
**

**Supplementary Figure 12 | Verification of the production of EML patterns without severe PR impurity residues.** **a** Schematic of PR stripping of green EML in the SPN structure. **b** Photographs of green EML patterns on a silicon wafer substrate (1 in^2^) before and after PR stripping under UV irradiation. **c** Large-area OM image of line-shaped (width: 3 µm; spacing: 4 µm) and circular (width: 4 µm; spacing: 4 µm) green EML patterns. The scale bar is 50 μm.


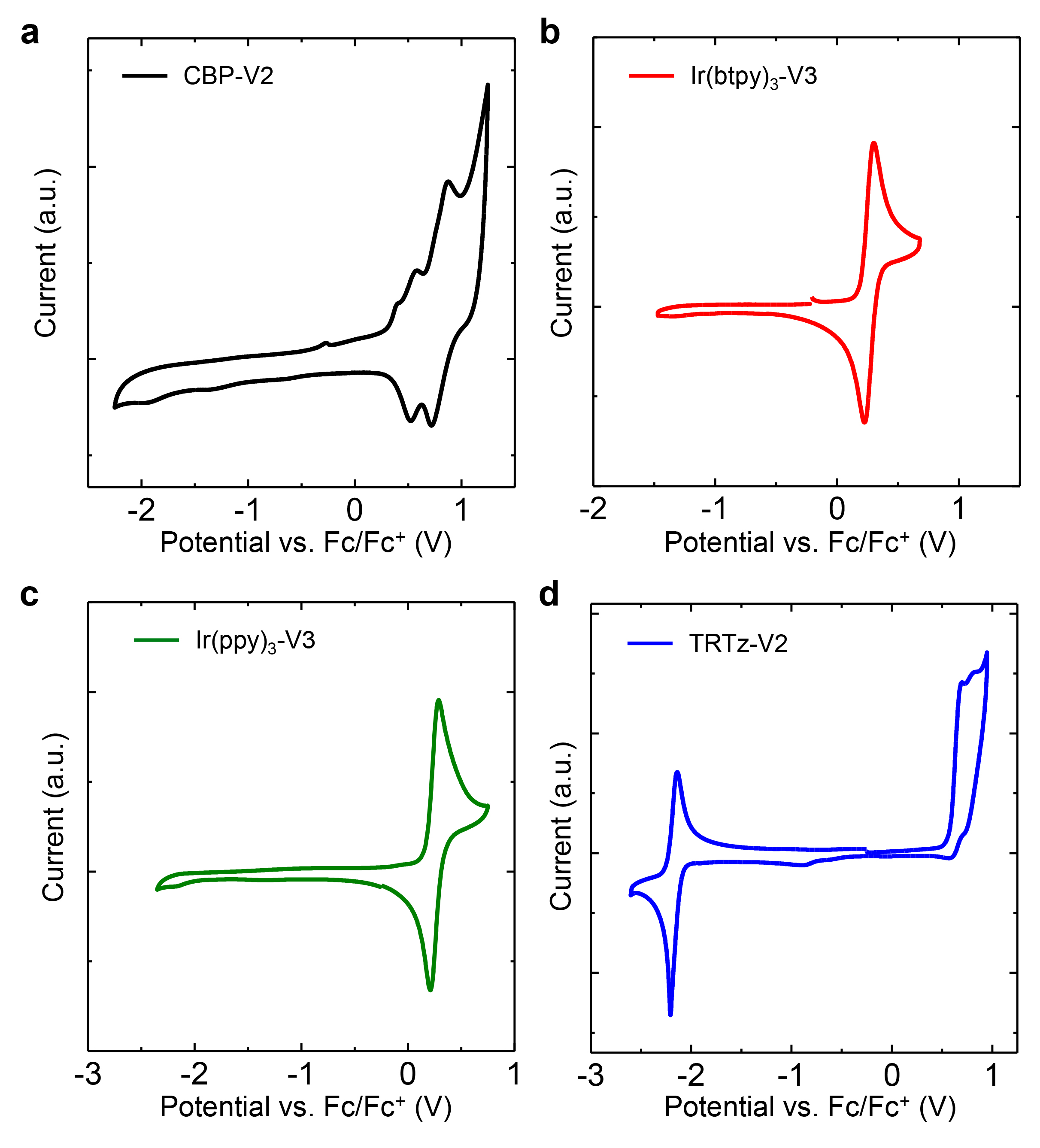


**Supplementary Figure 13 | Cyclic voltammogram of crosslinkable organic luminophores.** Cyclic voltammogram was measured for organic luminophores using 2:1 mixture of chlorobenzene and acetonitrile (10^-4^ M) as the solvent and tetra-n-butylammonium hexafluorophosphate (TBAPF_6_) as the supporting electrolyte. A ferrocenium/ferrocene (Fc/Fc^+^) couple was employed as the external reference^1^. The Cyclic voltammogram of **a** host, **b** red emitter, **c** green emitter, and **d** blue emitter.


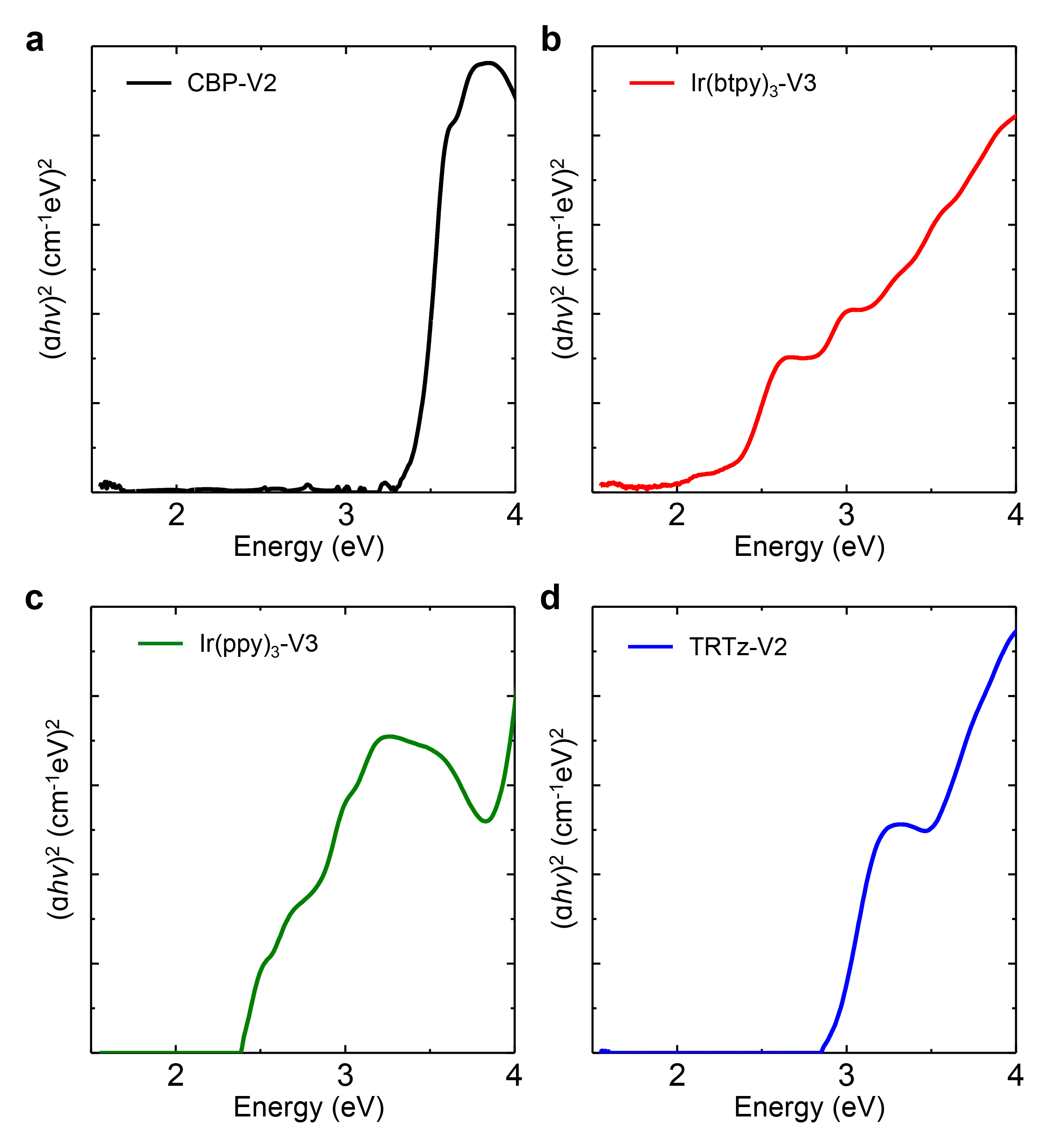


**Supplementary Figure 14 | Tauc plot of crosslinkable organic luminophore solutions.** The Tauc plot was calculated from the absorbance spectra of organic luminophore solutions in toluene (10^-5^ M)^2^. The Tauc plot of **a** host, **b** red emitter, **c** green emitter, and **d** blue emitter.

|  | ***V*_on_**  **(V)** | **EL peak**  **(nm)** | **EQE/CE/PE [%/cdA^-1^/lmW^-1^]** | | | ***L*_max_**  **(cdm^-2^)** | | **CIE**  **(x,y)** |
| --- | --- | --- | --- | --- | --- | --- | --- | --- |
|  |  |  | **@ Maximum** | **@ 100 cd/m^2^** | **@ 1000 cd/m^2^** |  |  | **@ 6 V** |
| Green | 3.7 | 524 | 4.8/17.5/12.4 | 4.8/17.3/10.0 | 4.4/16.0/6.9 | 7581 | (0.35, 0.61) | |
| Red | 3.9 | 604 | 4.9/6.5/5.1 | 4.1/5.3/2.8 | 1.9/2.3/0.7 | 1233 | (0.64, 0.36) | |
| Blue | 4.9 | 460 | 1.6/2.0/0.9 | 1.5/2.0/0.9 | 1.4/1.7/0.5 | 1866 | (0.17, 0.17) | |

**Supplementary Table 2 |** EL characteristics of red/green/blue (R/G/B) OLEDs based on EML in SPN structure.

*V*_on_, turn-on voltage; EL, electroluminescence; EQE, external electroluminescence quantum efficiency; CE, current efficiency; PE, power efficiency; *L*_max_, maximum luminance; CIE, Commission Internationale de l'éclairage.

**Supplementary Table 3 |** Comparison of EL characteristics of green OLEDs from this study and previous studies.

|  | *V*_on_ (V) | EL peak (nm) | *L*_max_ (cdm^-2^) | Maximum EQE (%) |
| --- | --- | --- | --- | --- |
| Our work | **3.7** | **524** | **7581** | **4.8** |
| [3] | 6.0 | 564 | 914 | 0.37 |
| [4] | 9.0 | (PL peak: 519 nm) | 1120 | 4.38 |
| [5] | 3.4 | 508 | 8700 | 3.05 |
| [6] | 7.0 | 525 | 400 | 2.0 |

*V*_on_, turn-on voltage; EL, electroluminescence; *L*_max_, maximum luminance; EQE, external electroluminescence quantum efficiency.

**Supplementary Table 4 |** Comparison of EL characteristics of red OLEDs from this study and previous studies.

|  | *V*_on_ (V) | EL peak (nm) | *L*_max_ (cdm^-2^) | Maximum EQE (%) |
| --- | --- | --- | --- | --- |
| Our work | **3.9** | **604** | **1233** | **4.9** |
| [7] | 5.32 | 666 | 1499 | 4.59 |
| [8] | 8.7 | 600 | 150 | 0.37 |

*V*_on_, turn-on voltage; EL, electroluminescence; *L*_max_, maximum luminance; EQE, external electroluminescence quantum efficiency.

**Supplementary Table 5 |** Comparison of EL characteristics of blue OLEDs from this study and previous studies.

|  | *V*_on_ (V) | EL peak (nm) | *L*_max_ (cdm^-2^) | Maximum EQE (%) |
| --- | --- | --- | --- | --- |
| Our work | **4.9** | **460** | **1866** | **1.6** |
| [9] | 6.5 | 415 | 50 | 0.47 |

*V*_on_, turn-on voltage; EL, electroluminescence; *L*_max_, maximum luminance; EQE, external electroluminescence quantum efficiency.


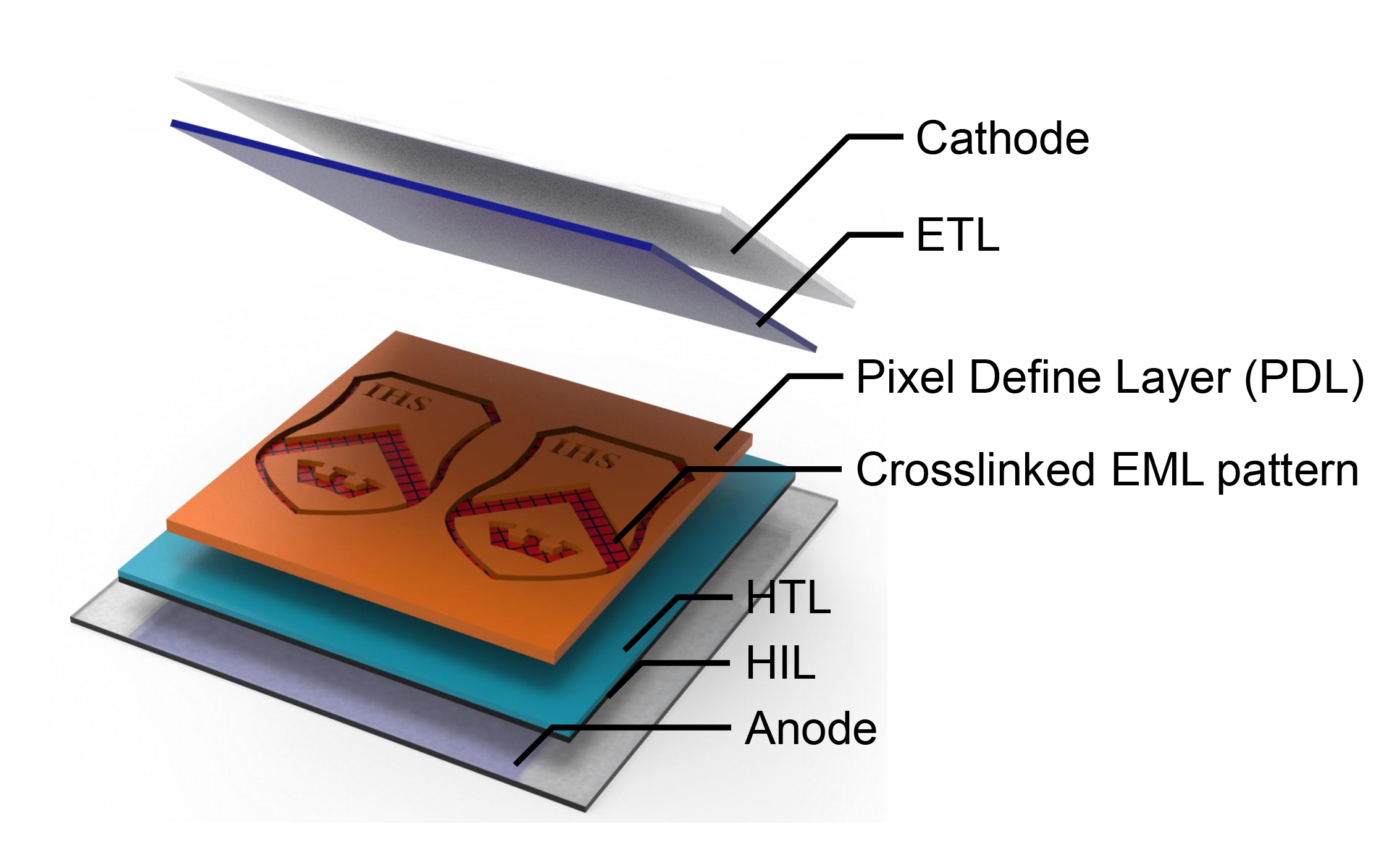


**Supplementary Figure 15 | Device structure scheme of the emissive layer-patterned OLED.** The pixel define layer (PDL) was formed using the KL5301 photoresist after emissive layer patterning.


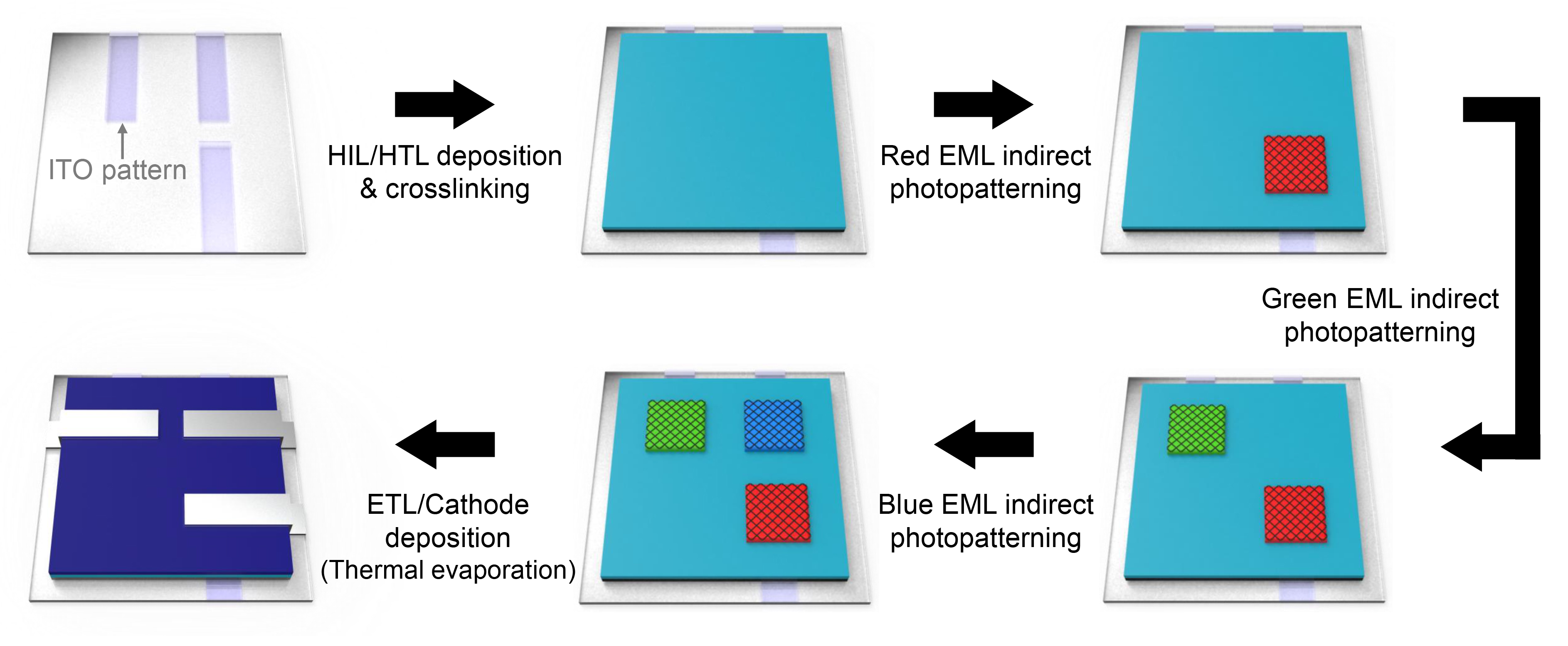


**Supplementary Figure 16 | Fabrication process of OLEDs with individual R/G/B color emission patterns.** R/G/B emissive layer patterns were formed through the repetition of the patterning method.


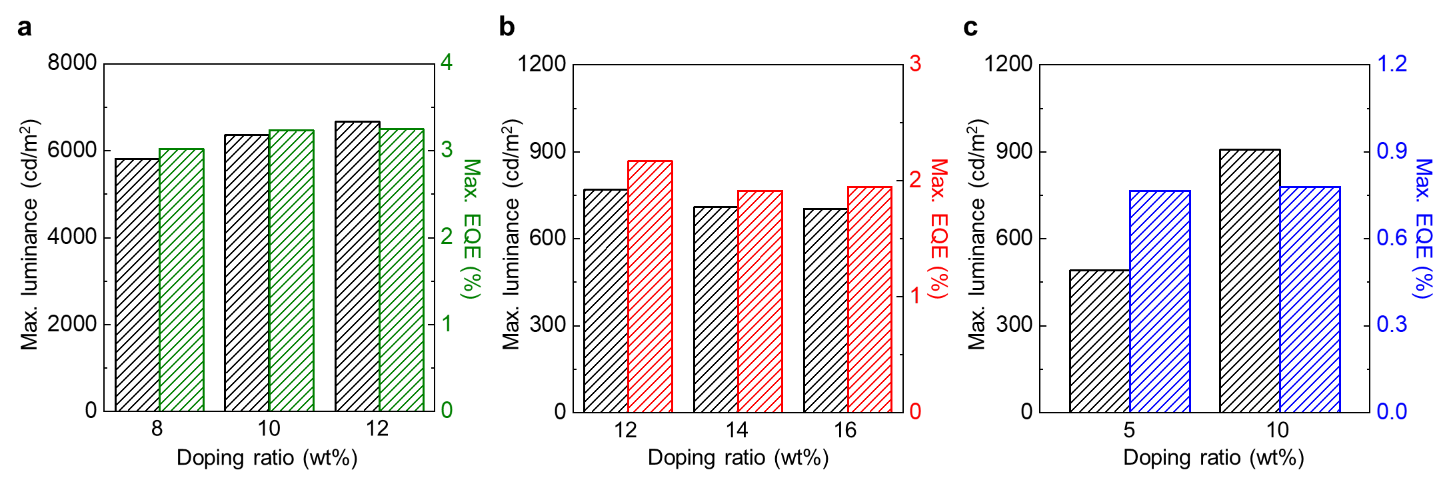


**Supplementary Figure 17 | Optimization of the doping ratio in the green/red/blue emissive layers of OLEDs.** Maximum luminance and maximum external quantum efficiency (EQE) of the **a** green, **b** red, and **c** blue OLEDs with different doping ratios. We used the EL properties as the criteria for determining the optimal ratio. First, we determined the reference doping ratio based on PL measurements, selecting the minimal ratio at which the PL peak of the host material disappears. Using this ratio as the starting point, we then searched for the optimal dopant ratio that produced the best EL characteristics (including both luminance and EQE) by varying the doping ratio around the reference ratio. Once we identified the optimal doping ratio, we further optimized the thickness of HTL and ETL to maximize device performance (This is why the results in **Supplementary Figure 17** are inferior to the final data presented in the manuscript).


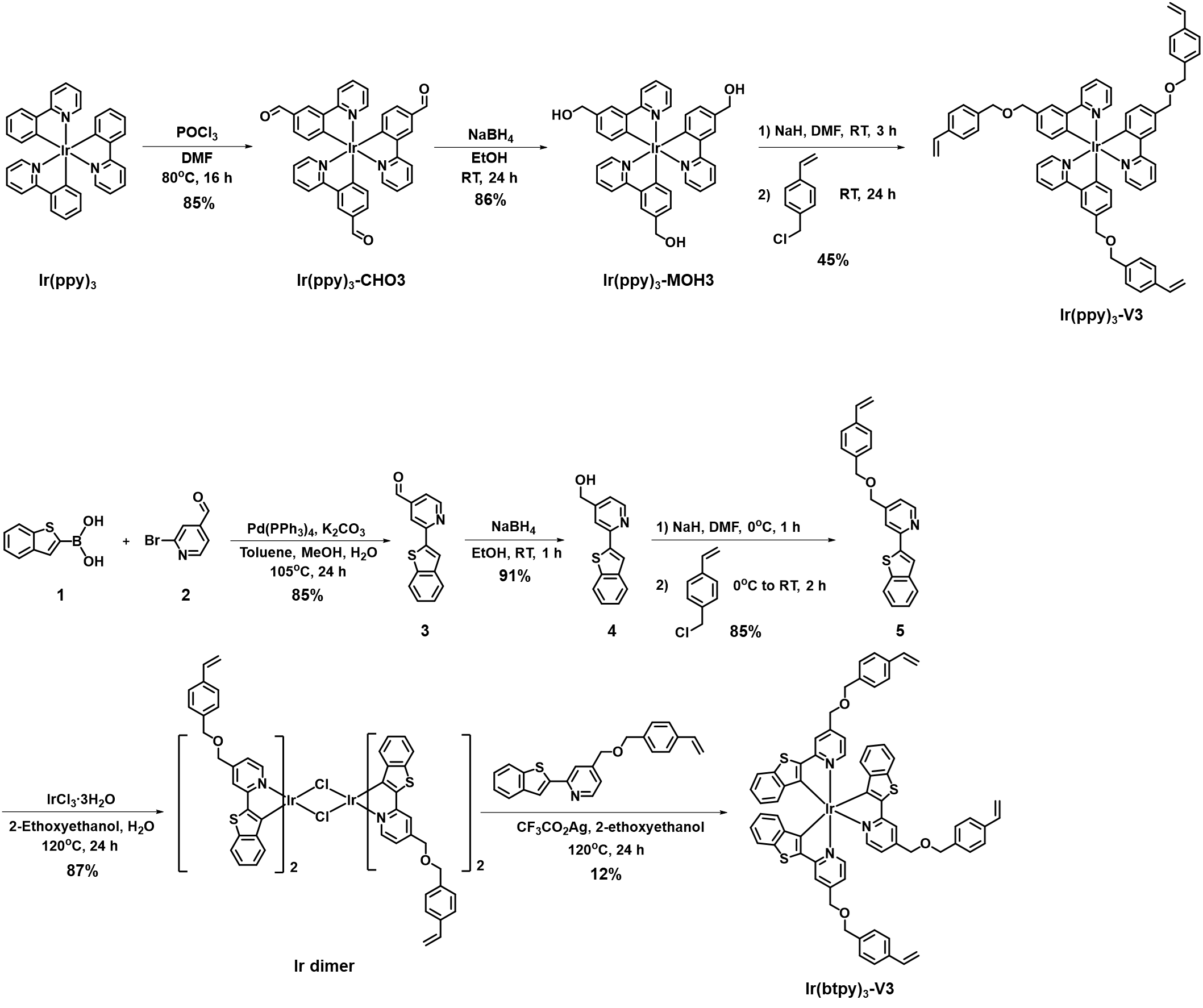


**Supplementary Figure 18 | Synthesis of Ir(ppy)_3_-V3 and Ir(btpy)_3_-V3.**


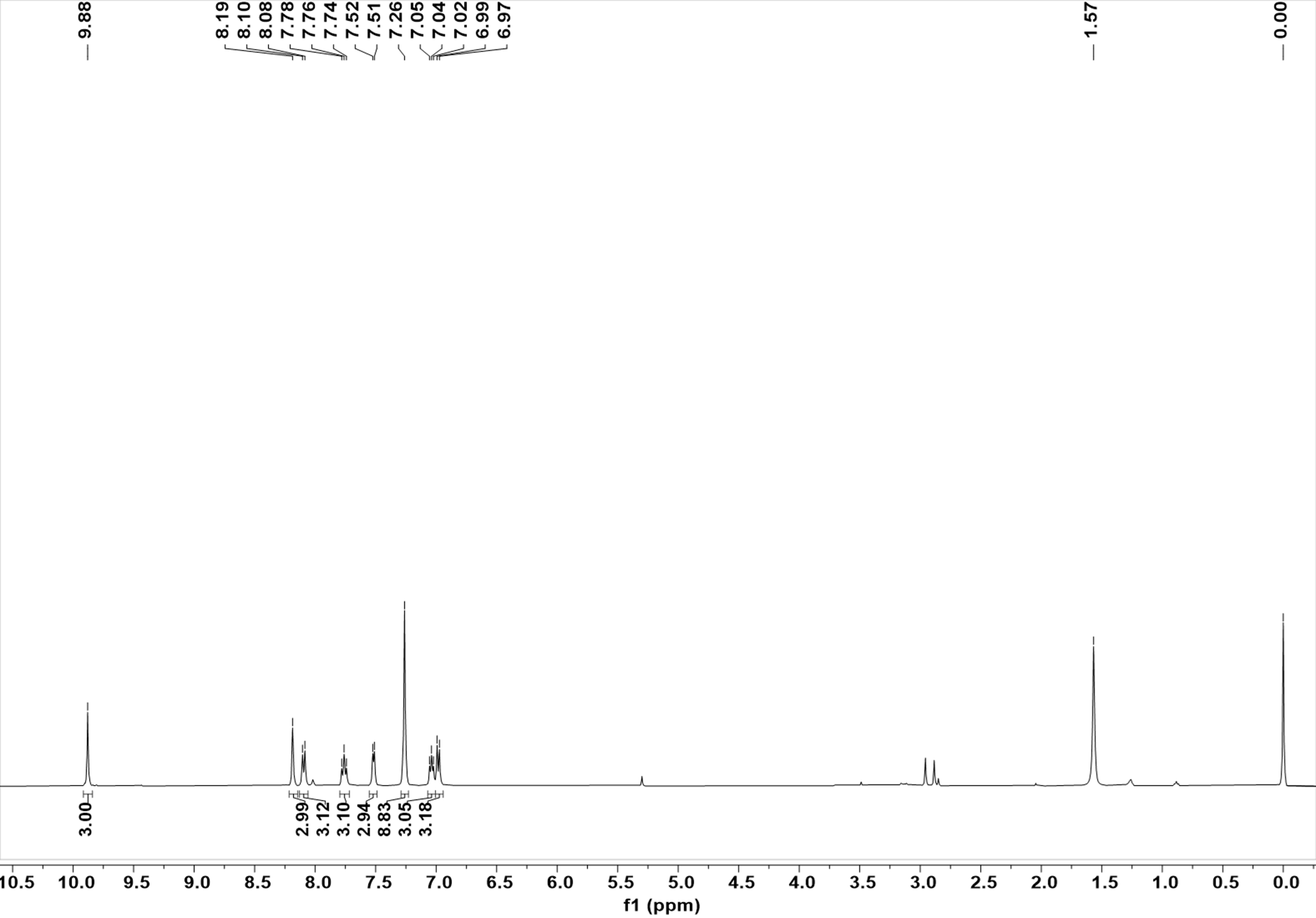


**Supplementary Figure 19 | ^1^H-NMR spectrum of Ir(ppy)_3_-CHO3.**


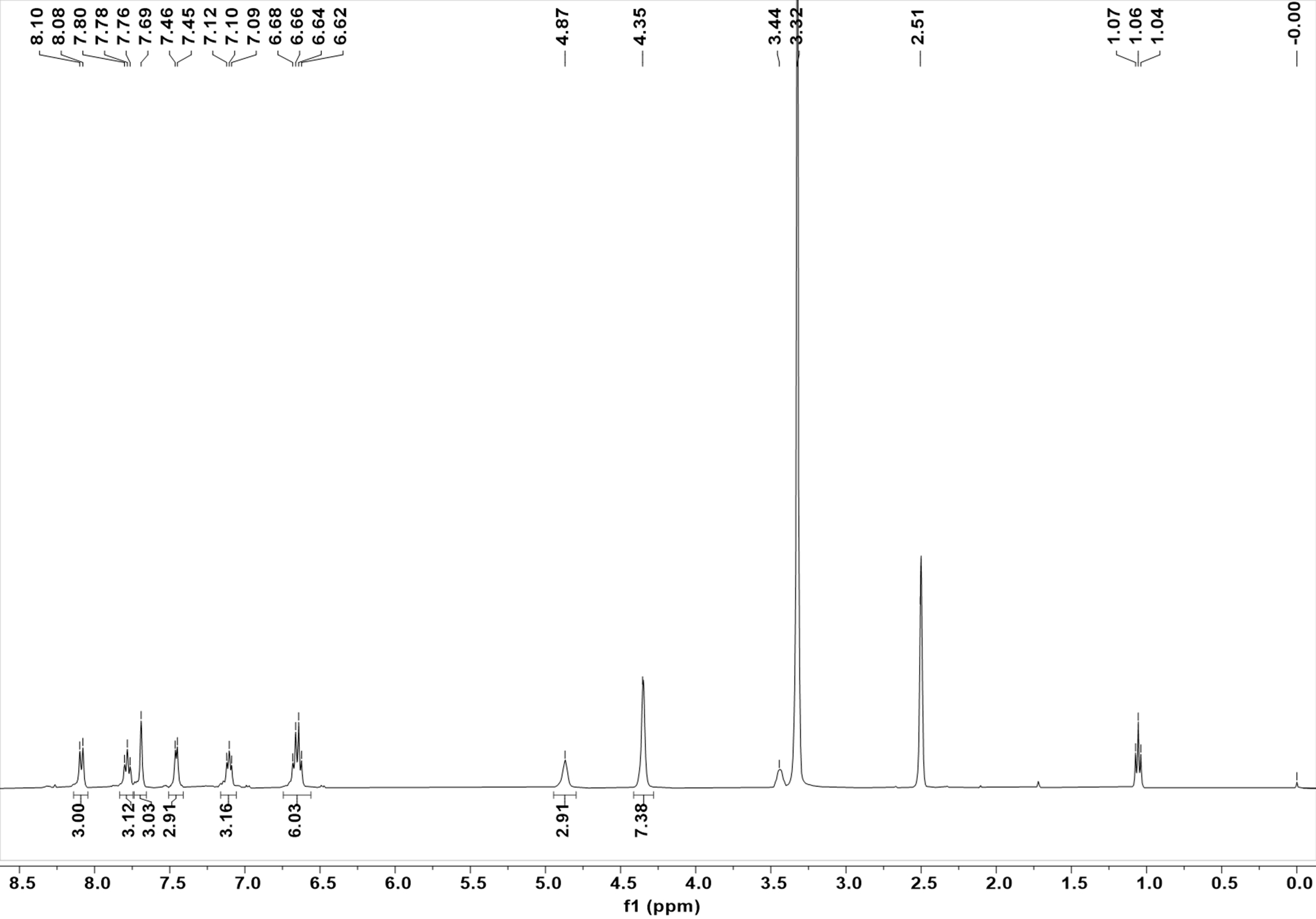


**Supplementary Figure 20 | ^1^H-NMR spectrum of Ir(ppy)_3_-MOH3.**

**Supplementary Figure 21 | ^1^H-NMR spectrum of Ir(ppy)_3_-V3.**

**Supplementary Figure 22 | ^13^C-NMR spectrum of Ir(ppy)_3_-V3.**


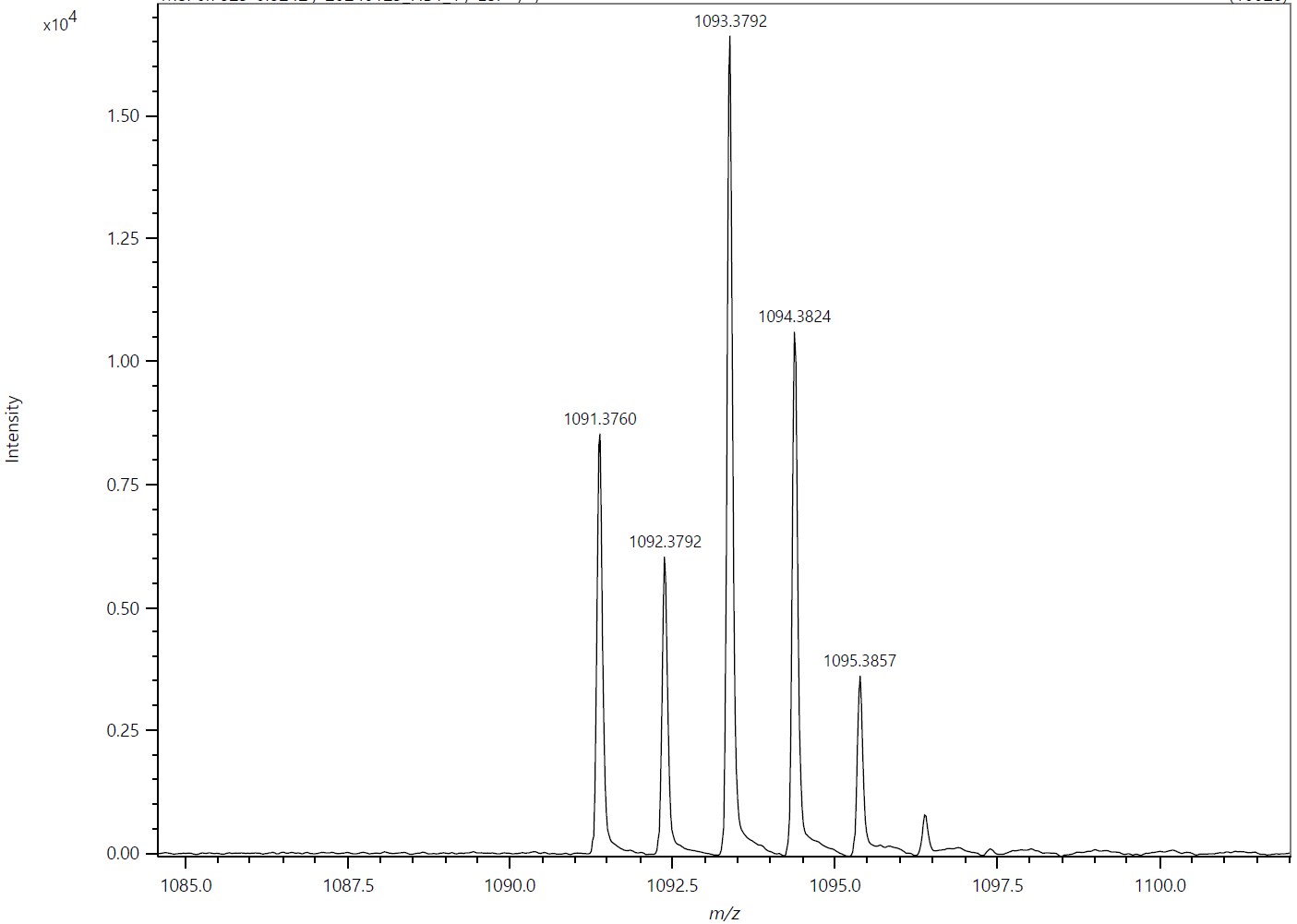


**Supplementary Figure 23 | ESI-MS spectrum of Ir(ppy)_3_-V3.**

**Supplementary Figure 24 | ^1^H-NMR spectrum of compound 3.**

**Supplementary Figure 25 | ^13^C-NMR spectrum of compound 3.**


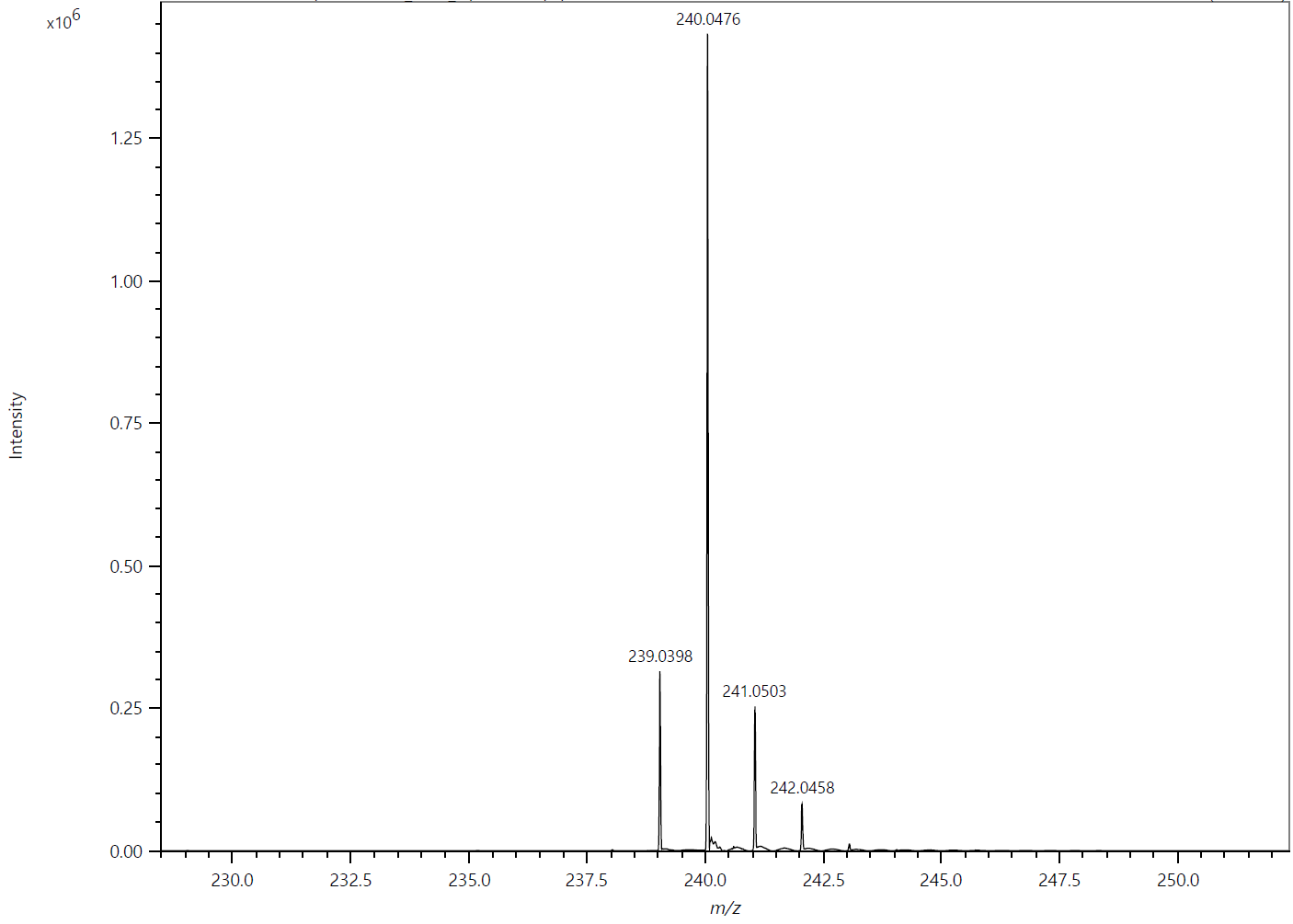


**Supplementary Figure 26 | ESI-MS spectrum of compound 3.**


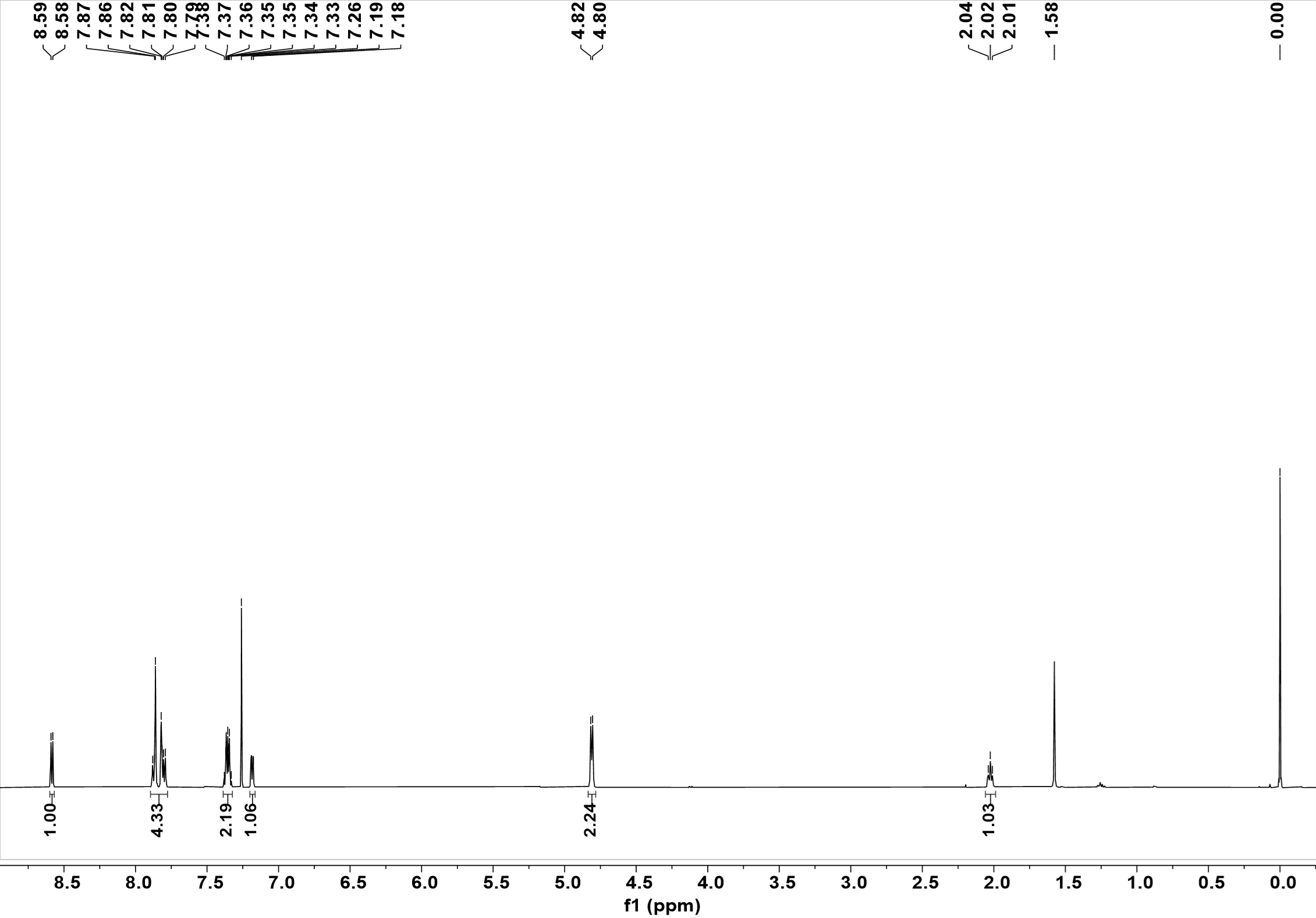


**Supplementary Figure 27 | ^1^H-NMR spectrum of compound 4.**

**Supplementary Figure 28 | ^13^C-NMR spectrum of compound 4.**


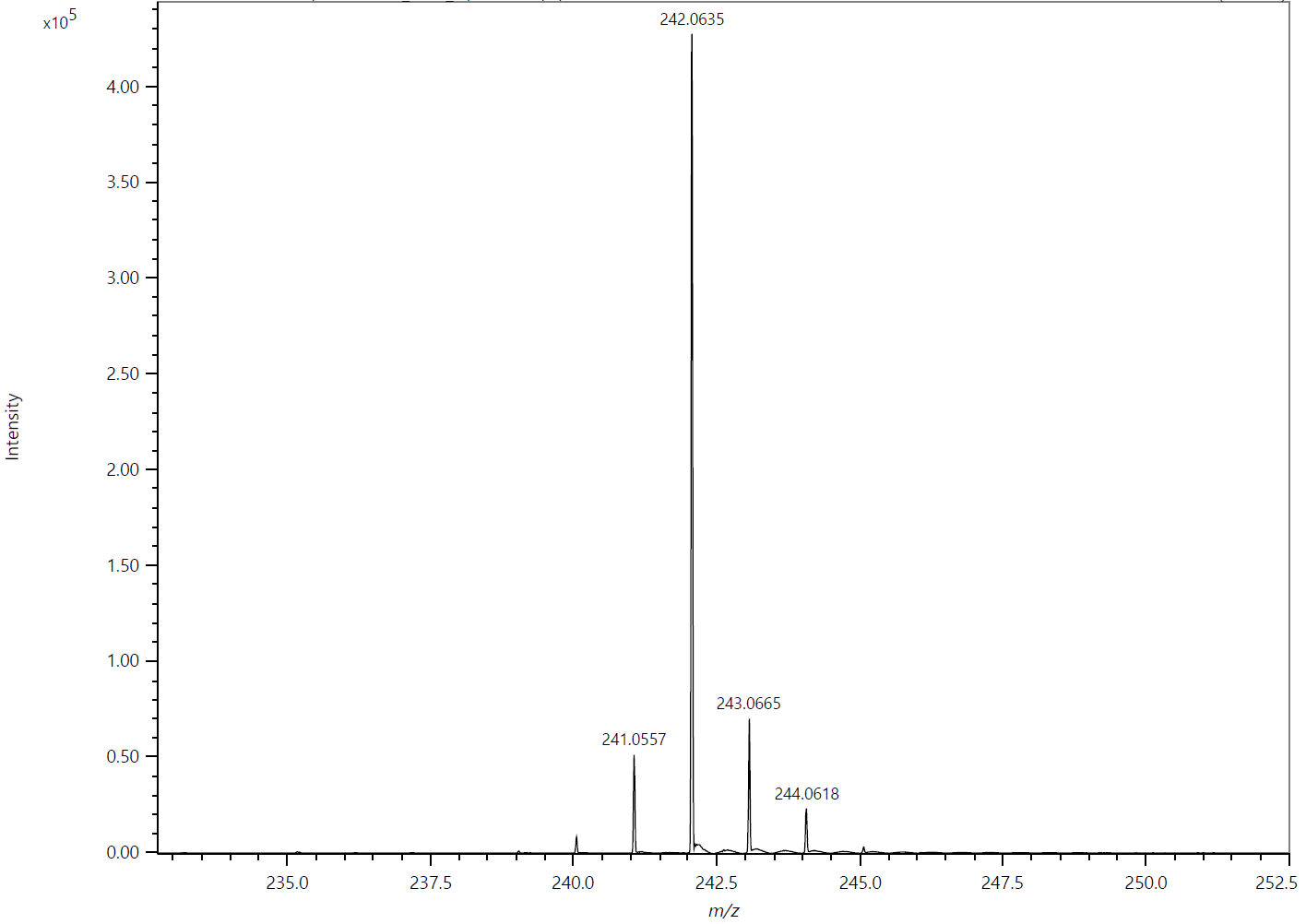


**Supplementary Figure 29 | ESI-MS spectrum of compound 4.**

**Supplementary Figure 30 | ^1^H-NMR spectrum of compound 5.**

**Supplementary Figure 31 | ^13^C-NMR spectrum of compound 5.**


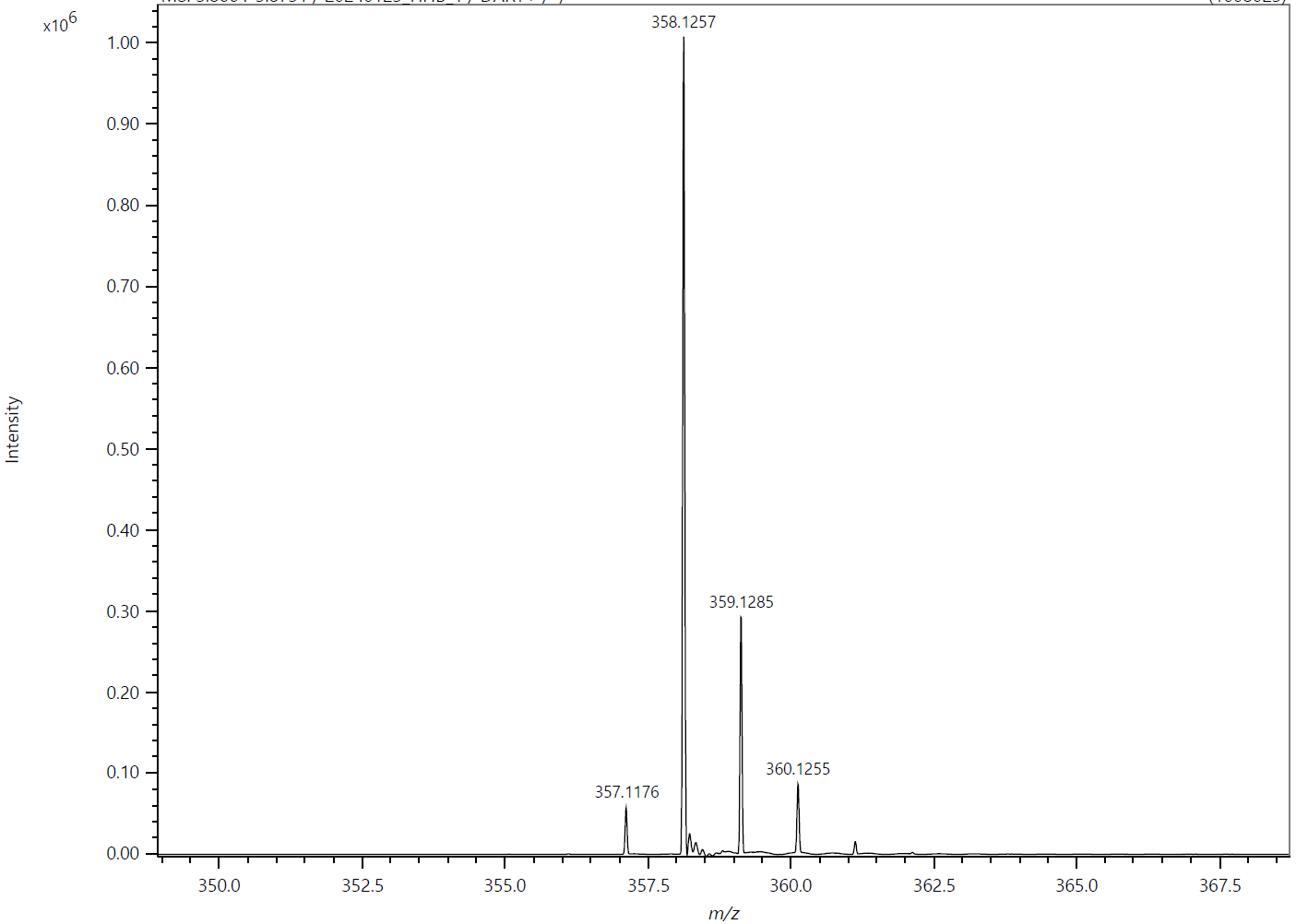


**Supplementary Figure 32 | ­ESI-MS spectrum of compound 5.**

**Supplementary Figure 33 | ^1^H-NMR spectrum of Ir(btpy)_3_-V3.**


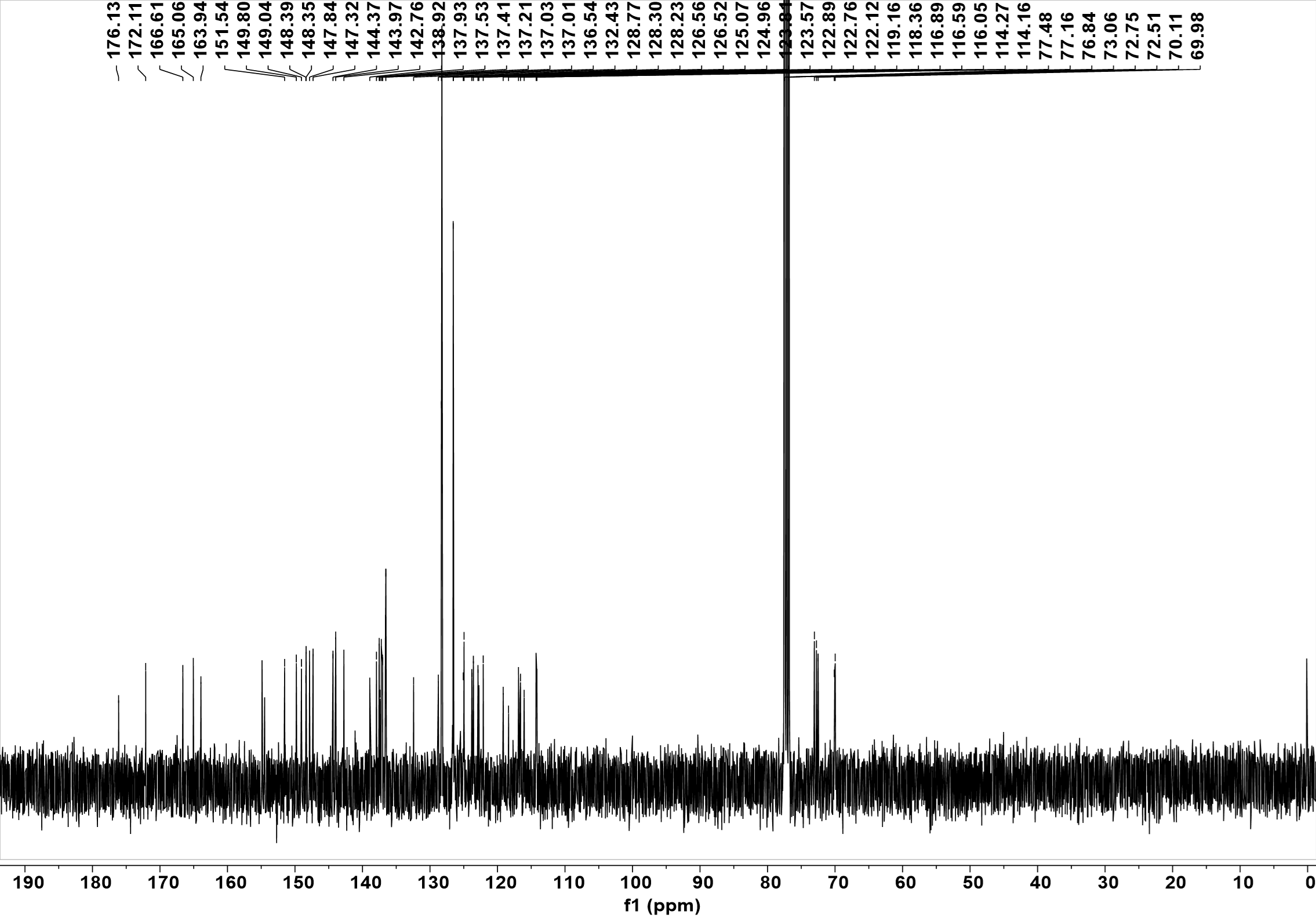


**Supplementary Figure 34 | ^13^C-NMR spectrum of Ir(btpy)_3_-V3.**

**
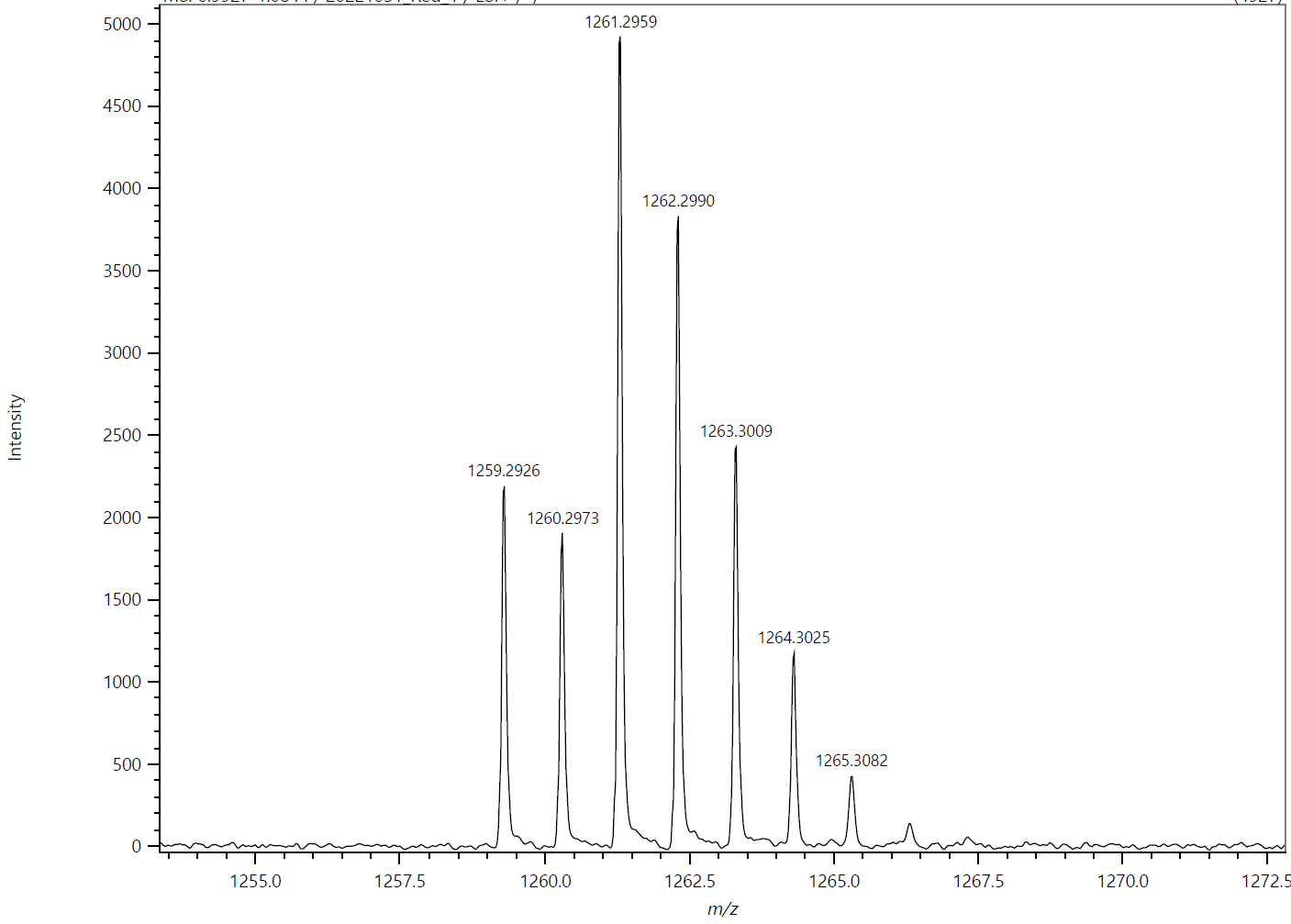
**

**Supplementary Figure 35 | ESI-MS spectrum of Ir(btpy)_3_-V3.**


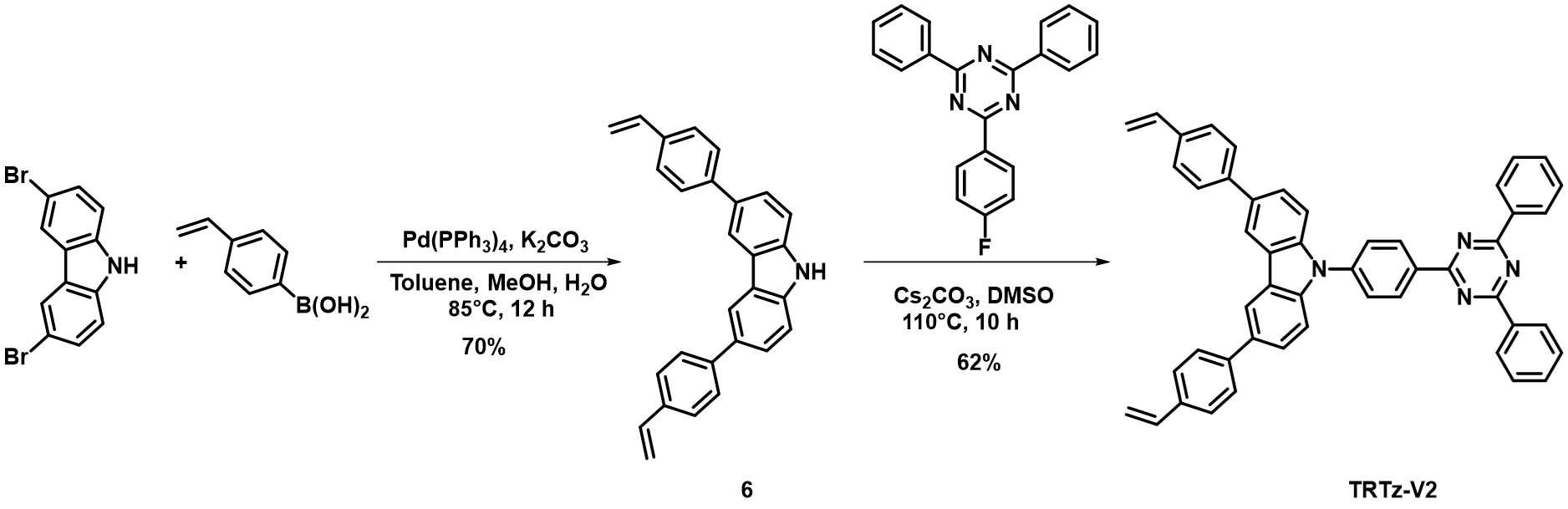


**Supplementary Figure 36 | Synthesis of TRTz-V2.**


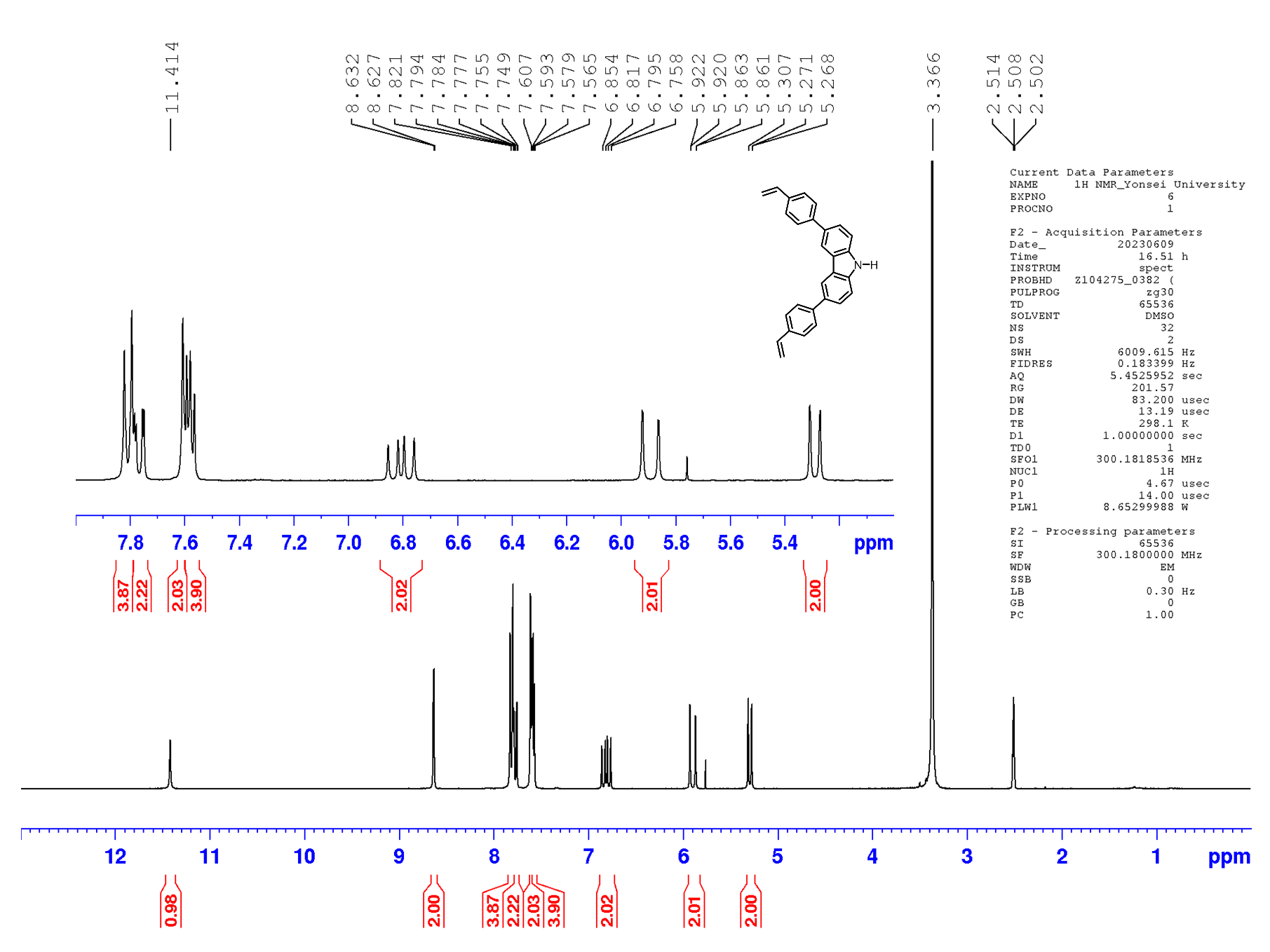


**Supplementary Figure 37 | ^1^H-NMR spectrum (300 MHz, DMSO-*d*_6_) of compound 6.**


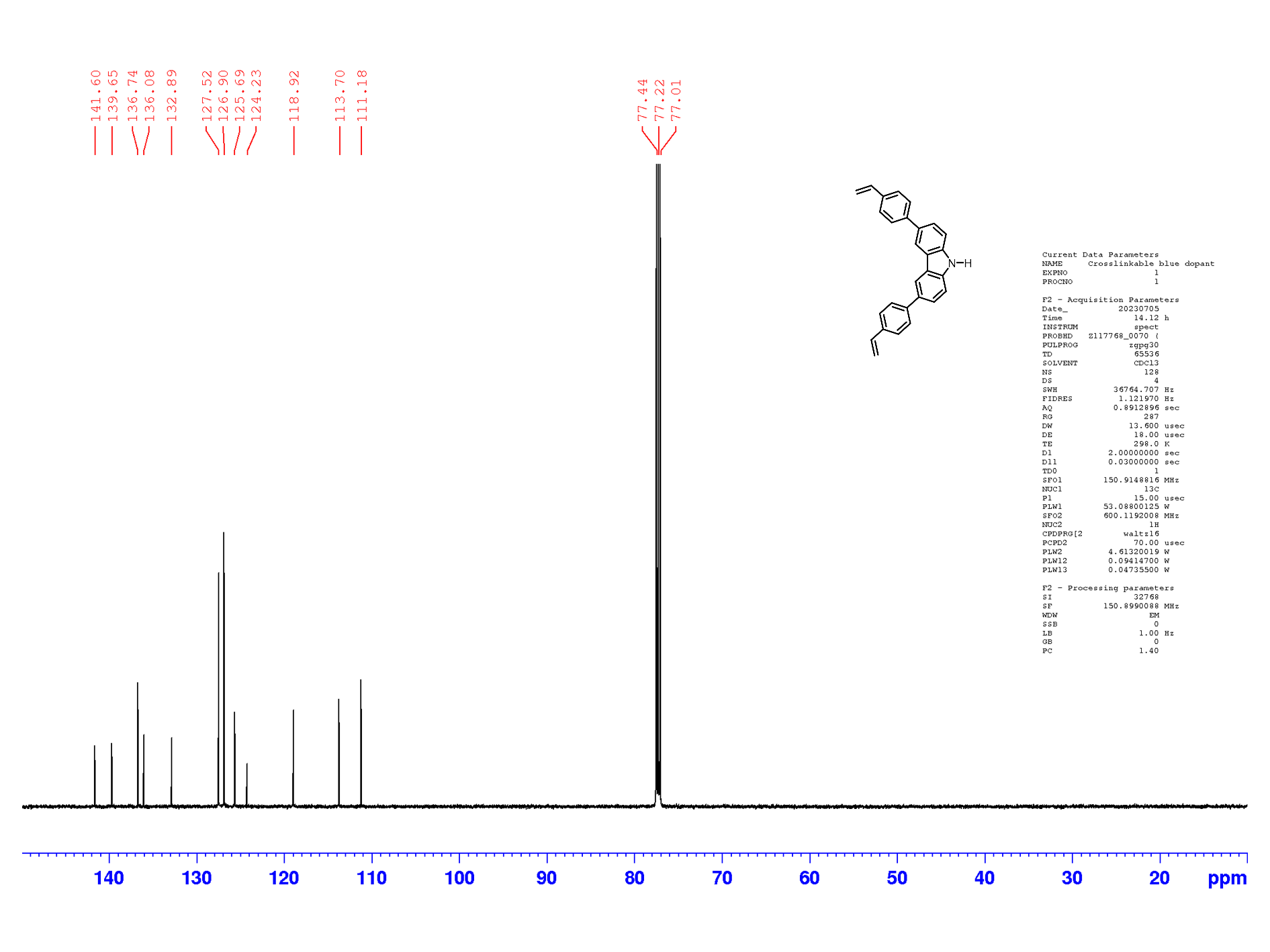


**Supplementary Figure 38 | ^13^C-NMR spectrum (150 MHz, CDCl_3_) of compound 6.**


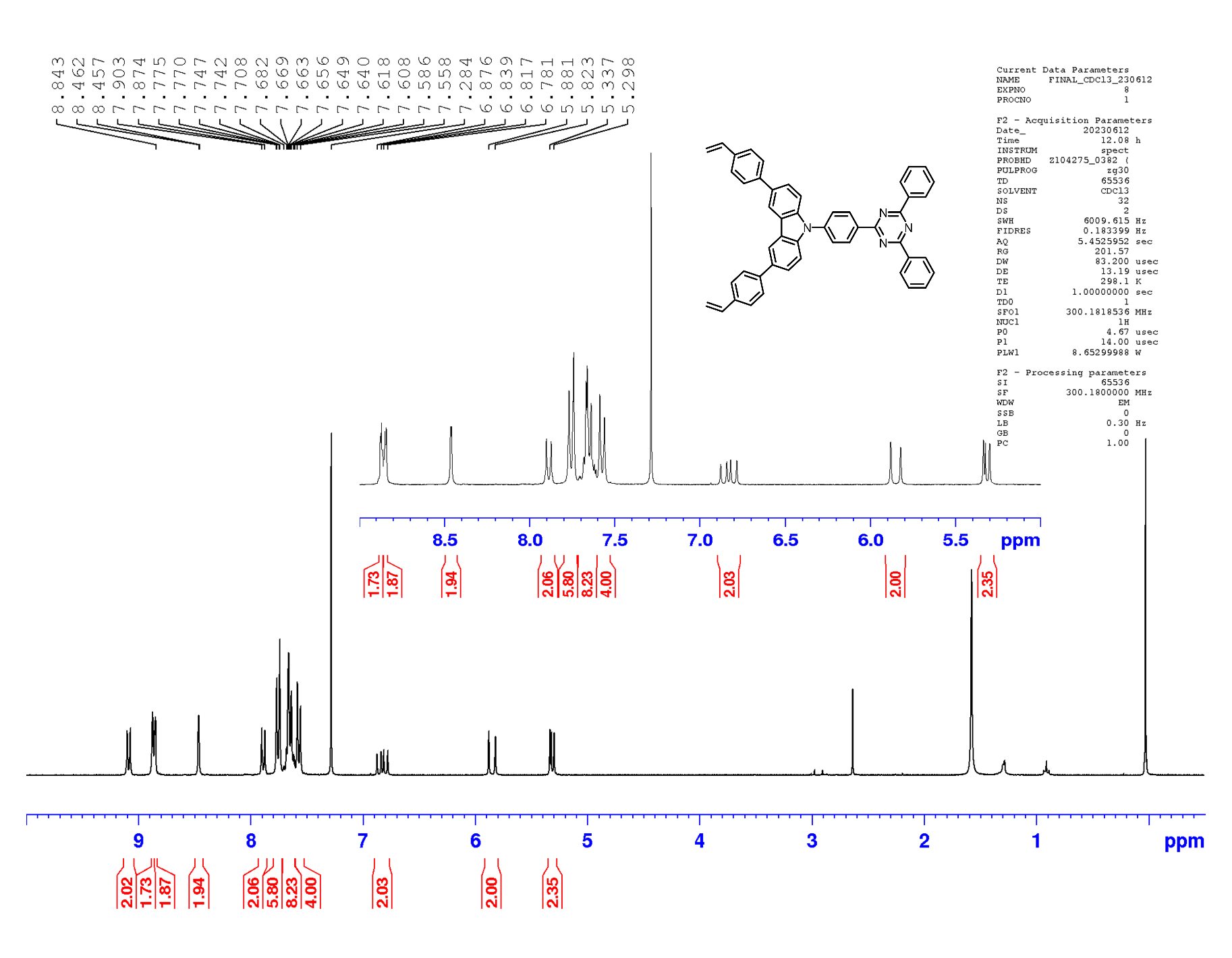


**Supplementary Figure 39 | ^1^H-NMR spectrum (300 MHz, CDCl_3_) of TRTz-V2.**


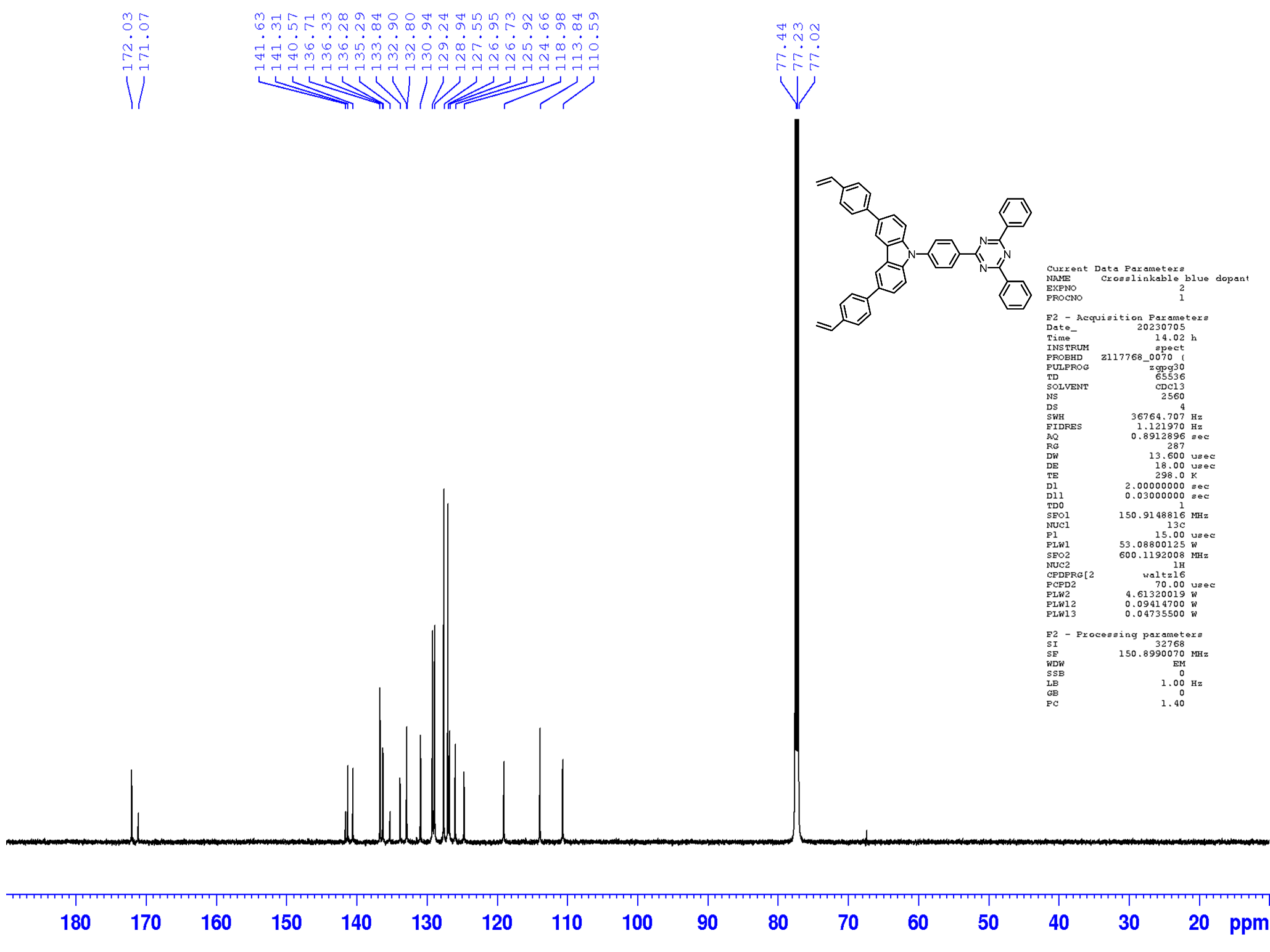


**Supplementary Figure 40 | ^13^C-NMR spectrum (150 MHz, CDCl_3_) of TRTz-V2.**

**References for Supplementary Information**

1. Adeniyi, A. A., Ngake, T. L. & Conradie, J. Cyclic Voltammetric Study of 2-Hydroxybenzophenone (HBP) Derivatives and the Correspondent Change in the Orbital Energy Levels in Different Solvents. *Electroanalysis* **32**, 2659-2668 (2020).

2. Duvenhage, M.-M.*, et al.* Determination of the optical band gap of Alq3 and its derivatives for the use in two-layer OLEDs. *Optical Materials* **42**, 193-198 (2015).

3. Liu, X.*, et al.* Synthesis and luminescence properties of two cross-linkable Ir(iii) complexes. *New Journal of Chemistry* **45**, 19154-19163 (2021).

4. Wu, S.*, et al.* Synthesis and luminescence properties of two Ir(iii) complexes containing styrene-modified phenylpyridine ligands. *New Journal of Chemistry* **45**, 3311-3318 (2021).

5. Wei, J.*, et al.* Enhanced performances of fully solution-processed OLEDs via introducing flexible chains into thermally cross-linked thermally activated delayed fluorescent materials. *Dyes and Pigments* **182**, 108624 (2020).

6. Ma, B., Kim, B. J., Poulsen, D. A., Pastine, S. J. & Fréchet, J. M. J. Multifunctional Crosslinkable Iridium Complexes as Hole Transporting/Electron Blocking and Emitting Materials for Solution-Processed Multilayer Organic Light-Emitting Diodes. *Advanced Functional Materials* **19**, 1024-1031 (2009).

7. Cho, W., Reddy, S. S., Kim, J., Cho, Y.-R. & Jin, S.-H. All solution-processed red organic light-emitting diode based on a new thermally cross-linked heteroleptic Ir(iii) complex. *Journal of Materials Chemistry C* **6**, 11714-11721 (2018).

8. Derue, L.*, et al.* All-Solution-Processed Organic Light-Emitting Diodes Based on Photostable Photo-cross-linkable Fluorescent Small Molecules. *ACS Applied Materials & Interfaces* **8**, 16207-16217 (2016).

9. Yang, J.*, et al.* Cross-linkable deep-blue small molecular material for solution-processed organic light-emitting diodes. *Optical Materials* **114**, 110945 (2021).
